# Isoniazid resistance profile and associated levofloxacin and pyrazinamide resistance in rifampicin resistant and sensitive isolates/from pulmonary and extrapulmonary tuberculosis patients in Pakistan: A laboratory based surveillance study 2015-19

**DOI:** 10.1371/journal.pone.0239328

**Published:** 2020-09-23

**Authors:** Sabira Tahseen, Faisal Masood Khanzada, Alamdar Hussain Rizvi, Mahmood Qadir, Aisha Ghazal, Aurangzaib Quadir Baloch, Tehmina Mustafa

**Affiliations:** 1 National TB Reference Laboratory, National TB Control Program, Islamabad, Pakistan; 2 Centre for International Health, Department of Global Public Health and Primary Care, University of Bergen, Bergen, Norway; 3 National TB Control Program, Ministry of National Health Services Regulation and Coordination, Islamabad, Pakistan; 4 Department of Thoracic Medicine, Haukeland University Hospital, Bergen, Norway; Jamia Hamdard, INDIA

## Abstract

**Background:**

Pakistan is among top five high burden countries for tuberculosis and drug resistant TB. Among rifampicin sensitive new pulmonary TB (PTB), prevalence of isoniazid resistance is 8.3% (95%CI: 7.0–10.7) and resistance to fluoroquinolone is higher (11·1%, 95%CI: 7·8–14·3) than isoniazid resistance.

**Method:**

Five year retrospective data (2015–2019) of drug susceptibility testing (DST) for *Mycobacterium tuberculosis* isolates, performed using recommended phenotypic (pDST) and/or genotypic (gDST) methods was analyzed stratified by rifampicin results for isoniazid resistance profiles and associated levofloxacin and pyrazinamide resistance.

**Findings:**

DST data was analyzed from 11045 TB patients. Isolates were tested using pDST (87%), gDST (92%) and both methods (79.5%). For both rifampicin and isoniazid, a significant difference (*P* < .001) was noted between resistance detected by pDST and gDST. Among isolates, tested by both methods (8787), 49% were resistant to rifampicin and 51.7% to isoniazid with discordance in resistant results of 15.8% for each, with 13.2% (570) of rifampicin resistance reported sensitive by pDST and 14.2% (660) of isoniazid resistance missed by gDST. Estimated isoniazid resistance among rifampicin sensitive new PTB, extrapulmonary TB and previously treated PTB was 9.8% (95%CI: 8.7–11.1), 6.8% (95%CI: 5.4–8.5) and 14.6% (95%CI: 11.8–17.9) respectively. Significant differences were reported between the genotypic profile of isoniazid resistance associated with rifampicin-resistant and sensitive isolates including detectable mutations (87% vs 71.6%), frequency of *inhA (*7.6% and 30.2%) *and katG* mutations (76.1% vs 41.2%) respectively. Among rifampicin resistant and sensitive isolates, a significantly higher level of resistance to levofloxacin and pyrazinamide was seen associated with isoniazid resistance.

**Conclusion:**

There are risks and many challenges in implementing WHO recommended treatment for isoniazid resistant tuberculosis. The laboratory based surveillance can complement random surveys in country specific planning for TB diagnostics and appropriate treatment regimens.

## Introduction

Isoniazid (INH) is one of the most important first-line medicines for the treatment of active tuberculosis (TB) with high bactericidal activity and a good safety profile [1–3]. Together with rifampicin (RMP), the two drugs represent the cornerstone of World health organization (WHO) recommended first-line treatment regimen used worldwide [4]. INH is also used in high dose in short course second line treatment regimens for drug resistant TB (DRTB) [5]. INH is critical not only for the treatment of active TB, but it is also highly effective in preventing disease and is the most commonly used medicine for latent TB infection [6]. INH resistance can thus undermine the effectiveness of treatment for both TB disease and infection.

Culture-based phenotypic testing is the current reference method for testing anti-TB medicines. It relies on testing at critical concentrations of drugs, that is, the lowest concentration of an anti-TB medicine that inhibits the in vitro growth of 99% of phenotypically wild-type strains of *Mycobacterium tuberculosis* (Mtb) [7]. In 2008, WHO endorsed the first genotypic drug susceptibility testing (DST) method for the rapid detection of multidrug-resistant (MDR) TB. INH resistance has been associated with multiple genes, most frequently *katG* and *inhA* [8–11]. The reported frequency of mutations varies in different geographical regions [11]. A number of studies have reported that the mutation at codon 315 in *katG* gene is often associated with a high level of INH resistance whereas mutations in *inhA* gene are associated with low-level resistance [8, 9].

Globally there are an estimated 10 million incident TB cases and among these, 7.0 million were notified in 2018. A high treatment success rates of at least 85% for new TB cases is regularly reported by all countries [7]. The estimated global prevalence of INH resistance among RMP sensitive new and previously treated TB (RsHr-TB) is 7.4% (95%CI: 6.5%–8.4%) and 11.4% (95%CI:9.4%–13.4%) respectively [12]. People infected with a TB strain that is resistant to INH, are reported to have a higher rate of unfavorable treatment outcomes with standard first line treatment [13].

In 2019, WHO issued guidelines recommending a modified 6-month treatment regimen containing RMP, ethambutol (EMB), pyrazinamide (PZA) and levofloxacin (LFX) for people with RsHr-TB. Exclusion of resistance to RMP is strongly recommended and empirical treatment is not advised. Fluoroquinolone (FQ) and PZA testing prior to treatment, is also advised where possible in order to prevent the acquisition of additional drug resistance. In addition, information on the specific INH mutations (*katG or inhA*) and overall host acetylator status at country or regional level are considered useful for regimen design [5].

Pakistan is country in South Asia with 212 M population and is among top five high burden countries (HBC) for TB and drug resistant TB (DRTB) [7]. TB disease burden is estimated at 562K incident TB cases at 265 /100K population. DOTs is the official strategy for TB control in the country and six month treatment regimen containing RMP throughout was adopted starting from 2012. Treatment success rate is maintained above 90% for new TB patients. In 2018, 64% of the estimated TB cases were notified including 80% pulmonary TB (PTB) [7]. First population based drug resistance survey (DRS) for smear positive PTB patients was conducted in 2012–13 and prevalence of RMP resistance was estimated at 4.2% and MDR at 3.7% in new cases. Any resistance to INH not associated with RMP resistance (RsHr-TB) was reported in 8.3% (95%CI: 7.0–10.7) of new and 7.1% (95%CI: 4.0–11.4) in previously treated PTB patients [14]. Subsequently, DRS isolates were also tested for FQ and PZA resistance among RMP resistant and sensitive population as part of a multicounty surveillance project and FQ resistance was reported respectively in 21·8% (95%CI;13·1–30·5)and 11·1%(95%CI:7·8–14·3) and PZA resistance in 39.5% (30.1–48.9) and 0.5%(0.1–0.8) [14, 15]. However there is limited published data on molecular markers for INH resistance [12]. We analyzed routine laboratory data to study diagnostic and clinical implications of the prevalent phenotypic and genotypic profile of INH resistance and LFX and PZA resistance associated with INH resistance in RMP resistant (RrHr) and sensitive (RsHr) isolates from pulmonary and extrapulmonary TB (EPTB) patients.

## Study setting, design and methodology

### Study setting

In Pakistan, Xpert MTB/RIF (Cepheid, Sunnyvale, CA, USA) testing services are decentralized, patient reported to have RMP resistance are referred to specialized DRTB treatment sites and specimens are than referred for DST. Specimen transport is well established between DRTB treatment sites and culture and DST laboratories. National TB Reference laboratory (NTRL) is located in Islamabad, the federal capital of the country and receive clinical specimens or culture isolates, referred routinely from treatment sites across Punjab province and three territories including Islamabad, Azad Jammu Kashmir and Gilgit Baltistan together covering more than 50% of the country population. NTRL offer diagnostic and DST services for patients already diagnosed as RMP resistant by Xpert MTB/RIF or who are at risk of drug resistance or presumed to have TB specially PTB in children and EPTB.

### Study design

This is a retrospective five-year (January 2015-December 2019) laboratory based surveillance study. All confirmed Mtb isolates from patients having PTB or EPTB, tested either using phenotypic DST (pDST) and/or genotypic method (gDST) with DST results reported for both RMP and INH were included.

### Laboratory methods

Throughout the study period, gDST for RMP, INH and FQ was performed by Line Probe assay(LPA), using GenoType MTBDRplus and MTBDRsl version 2.0 (Hain Lifescience, Nehren, Germany). MGIT 960 automated system (BD, Sparks, MD, USA) was used to perform pDST at recommended critical concentrations for RMP (1.0ug/ml), INH (0.1ug/ml), PZA(100ug/ml) and ofloxacin (2ug/ml) during 2015–17 and LFX (1.0ug/ml) during 2018–19 [16].

All clinical samples were processed for culture and pDST was performed for all confirmed Mtb isolates. LPA was introduced in late 2015 and gDST was increasingly performed directly on clinical samples from known RMP resistant patients. Culture isolates were used for gDST for patients having RMP resistance with invalid results on direct testing, known RMP sensitive or with unknown RMP status. Sequencing was not performed as facilities were not established.

All recommended quality control measures for DST were followed during study period including, testing of known sensitive and resistant control strains with each batch of DST [17] and regular participation in annual external quality assessments conducted by WHO collaborating center (Supra National TB reference laboratory, ITM, Antwerp, Belgium) with successful certification for first and second line DST.

### Data management

Case based data was extracted from computerized laboratory information system of NTRL in CSV format. Data was then checked for duplications, for each patient only first pDST and /or gDST results reported either from same or paired samples were included. Duplicate or sequential DST results from same patient were excluded. After cleaning, all personal identifiers were removed before analysis.

### Data analysis

Data were analyzed using STATA1 v13.1 (StataCorp, 4905 Lakeway Drive, College Station, Texas 77845, USA). Mean, median and quartiles were analyzed for quantitative variables and 95% confidence intervals for comparisons between groups. Two sample proportion test was used to analyze differences in proportions between groups. A p-value of *<*0.05 was considered statistically significant.

Study population was analyzed for demographic characteristics of patients, previous history of TB treatment, province of residence, referring health facilities and AFB smear results. All phenotypic and genotypic results were first analyzed independently for proportion of RMP and INH resistance. Further analysis was done on large subset of Mtb isolates tested by both DST methods. LPA results were interpreted based on recommended guidelines [18]. Results were compared for agreement and discordance between two DST methods for RMP and INH. For final interpretation, resistance conferring mutations to RMP and INH were considered to be true resistance, even if phenotypic testing showed susceptibility. Based on the final interpretations, prevalence of INH and RMP resistance, genetic profile of INH resistance and associated resistance to LFX and PZA was analyzed stratified by RMP result, disease site and previous history of TB treatment. Genetic profiles of INH resistant isolates was studied for the frequency of mutations known to confer resistance among samples displaying either phenotypic and/or genotypic resistance to INH. The INH resistant isolates were sorted into four groups: Group1: phenotypic resistant but genotypic wild type (WT), Group 2: isolates with *inhA* mutation/s only, Group 3: isolates with *katG* mutation only and Group 4: isolates with combined *katG* and *inhA* mutations. Annual trends were analyzed for RsHr-TB, genetic profile of INH resistance and associated FQ and PZA resistance.

Patient having no or unknown previous history of ATT were categorized as new and those with history of previous treatment or on treatment for more than a month as previously treated. For estimation of INH resistance among previously treated, data was analyzed only from those patient with history of WHO recommended TB treatment for new TB patients. Annual trend for INH resistance was analyzed for years with DST results available by both methods for more than 100 patients. For FQ resistance, all strains reported resistant to OFX (2015–17), LFX (2018–19) or showing FQ conferring mutations on LPA were considered LFX resistant. Results were compared with the prevalence estimates of resistance reported in DRS [14] and primary drug resistance in EPTB [19].

### Ethics statement

The study protocol was approved by IRB of Common unit for HIV/AIDS, TB and Malaria program, Islamabad, Pakistan. The antimicrobial resistance was analyzed in Mtb strains isolated routinely in the laboratory for diagnostic purposes. To maintain confidentiality of the patients, de-identified data was used for analysis.

## Results

### Study population

**Fig 1:**
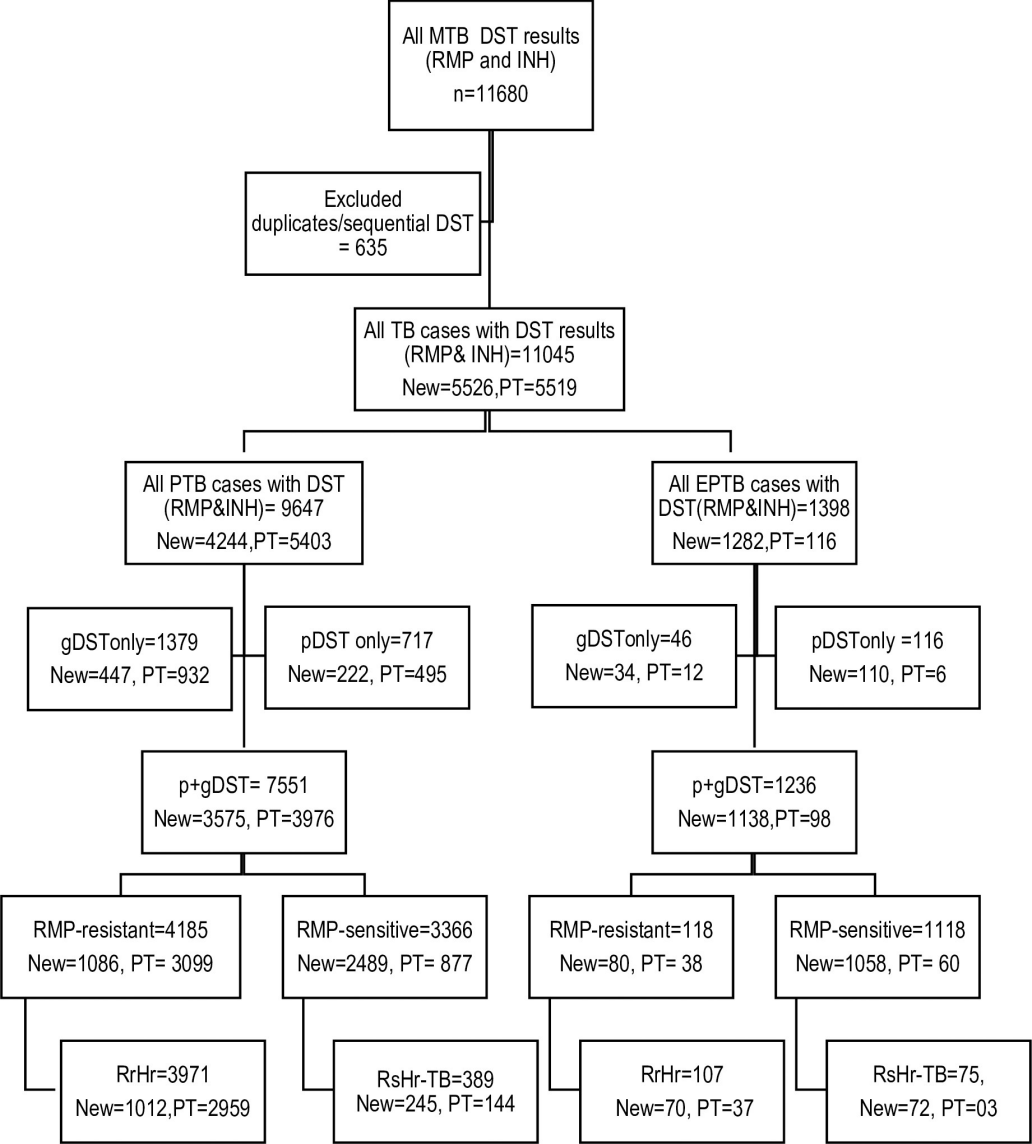
Flow diagram showing drug susceptibility testing of *Mycobacterium tuberculosis* isolates by disease site, history of TB treatment and DST method, National TB reference laboratory, Pakistan 2015–2019. RMP-rifampicin, INH-isoniazid, R-resistant, S-sensitive, pDST-phenotypic drug susceptibility testing; gDST-Genotypic drug susceptibility testing, Rr-rifampicin resistant, Rs-rifampicin sensitive, Hr-isoniazid resistant, Hs-Isoniazid sensitive.

During the study period (January 2015 to December 2019), altogether 11,680 DSTs were reported, 635 were duplicate and/or sequential DST, which were excluded, and final analysis was performed on 11,045 DST results from same number of patients (Fig 1). Patients included were referred from 86 health facilities in 29 districts. Altogether 65.8% patients were referred by tertiary care, 30.2% by secondary and 3.9% by primary health care facilities and 83.8% of patient referral was from health facilities managing DRTB (S1 Table).

Median age of TB patients was 30 years (32 years for PTB and 23 years for EPTB), 7.5% were children (<15yrs), 48.8% females and 86% were resident of Punjab province. Of all patients, 87.3% presented with PTB, 56% of PTB and 8.3% of EPTB patients had a history of previous TB treatment. Among previously treated PTB, 44.8% had history of TB treatment regimen for new patients, 11.8% for retreatment and 22.4% for DRTB (S1 Table). Details of pulmonary and extrapulmonary specimens processed for testing is given in S2 Table.

### Rifampicin and isoniazid resistance

#### Rifampicin and isoniazid resistance by DST methods

Of all 11045 TB patients, pDST results were available for 9620 including 14.1% (1352) EPTB, gDST for 10212 including 12.6% (1282) EPTB and both pDST and gDST results for 8787 patients including 14.1% (1236) EPTB. RMP and INH resistance detected by pDST and gDST is shown in Table 1 and annual trend in S3 Table. Among PTB isolates, a significant difference (*P* < .001) was seen between resistance detected by pDST and gDST for RMP (49.8 vs 56.4%) as well as INH (58.4 vs 49.2%) respectively but difference was not significant among EPTB isolates (Table 1).

**Table 1:** Isoniazid and rifampicin resistance detected by phenotypic and genotypic DST methods in Mtb isolates from pulmonary and extrapulmonary TB patients, National TB reference laboratory, Pakistan 2015–19.

	All TB (n = 11045)			Pulmonary TB (n = 9647)			Extrapulmonary TB (n = 1398)		
Isolates tested	pDST	gDST	p+gDST	pDST	gDST	p+gDST	pDST	gDST	p+gDST
	9620	10212	8787	8268	8930	7551	1352	1282	1236
**Any rifampicin resistance**									
RMP resistant-n	4225	5146	4303	4117	5027	4185	108	119	118
RMP-resistant-%	43.9%	50.4%	49.0%	49.8%	56.3%	55.4%	8.0%	9.3%	9.5%
(95%CI)	(42.9–44.9)	(49.4–51.4)	(47.9–50.0)	(48.7–50.9)	(55.2–57.3)	(54.3–56.5)	(6.6–9.6)	(7.7–11.0)	(8.0–11.3)
**Any isoniazid resistance**									
INH resistant-n	5025	4557	4542	4828	4393	4360	197	164	182
INH resistant-%	52.2%	44.6%	51.7%	58.4%	49.2%	57.7%	14.6%	12.8%	14.7%
(95%CI)	51.2–53.2	43.7–45.6	50.6–52.7	57.3–59.5	48.2–50.2	56.6–58.9	12.7–16.6	11.0–14.7	12.8–16.8
**Isoniazid resistance associated with rifampicin resistance (MDR)**									
RrHr/Rr-TB-n	4117/4225	4112/5146	4078/4303	4010/4117	4008/5027	3971/4185	107/108	104/119	107/118
RrHr/Rr-TB %	97.4%	79.9%	94.8%	97.4%	79.7%	94.9%	99.1%	87.4%	90.7%
(95%CI)	(96.9–97.9)	(78.8–81.0)	(94.1–95.4)	(96.0–97.9)	(78.6–80.8)	(94.2–95.5)	(94.9–100)	(80.0–92.8)	(83.9–95.2)
**Isoniazid resistance associated with rifampicin susceptible (RsHr)**									
RsHr/Rs-TB-n	908/5395	445/5066	464/4484	818/4151	385/3903	389/3366	90/1244	60/1193	75/1118
RrHr/Rs-TB-%	16.8%	8.8%	10.3%	19.7%	9.9%	11.6%	7.2%	5.2%	6.7%
(95%CI)	(15.8–17.9)	(8.0–9.6)	(9.5–11.3)	(18.5–20.9)	(8.9–10.8)	(10.5–12.7)	(5.9–8.8)	(1.7–6.8)	(5.4–7.2)

N = number isolates tested, n = number resistant, pDST = Phenotypic drug susceptibility testing, gDST = Genotypic drug susceptibility testing

#### Correlation between genotypic and phenotypic DST results

Of all isolates tested by both DST methods (n = 8787), an agreement in results was reported for both RMP and INH in 81% (7117), RMP in 92.3% (8109) and INH in 91.9% (8071) of isolates (Table 2; S3 and S4 Tables). A significant difference was seen between RMP resistance (*P* < .001) detected by pDST (42.5%, 95%CI; 41.5–43.5) and gDST (47.7%, 95%CI; 46.7–48.8) and INH resistance (*P* < .001) detected by pDST (51.1%, 95%CI; 50.0–52.1) and gDST (44.2%:95%CI 43.1–45.2). Compared to pDST proportion of INH resistance by gDST was significantly lower among RMP resistant (97.3 vs 83.1%) and RMP sensitive (16.9 vs 8.7%) isolates. Taking both DST results into account, altogether RMP resistance was detected in 4303 (49%, 95%CI; 47.9–50.0) isolates with 678 (15.8%) discordant results including 570(13.2%) reported sensitive by pDST and 108(2.5%) by gDST. INH resistance was detected in 4542(51.7%, 95%CI; 50.6–52.7) isolates with 716(15.8%) discordant result including 660(14.2%) reported sensitive by gDST and 56 (1.2%) by pDST. (Table 2; S4 Table).

**Table 2:** Correlation between phenotypic and genotypic drug susceptibility testing results for rifampicin and isoniazid in Mtb isolates from all type TB patients, National TB reference Laboratory Pakistan 2015–19.

		All	Rifampicin phenotypic(MGIT960) and genotypic(LPA) DST results							
			pRgR	pRgS	pSgR	pSgS	pRgNA	pSgNA	pNAgR	pNAgS
Isoniazid phenotypic(MGIT960)and genotypic (LPA) DST results	**All**	**11045**	**3625**	**108**	**570**	**4484**	**492**	**341**	**951**	**474**
	pRgR	**3826**	3097	66	352	311				
	pRgS	**660**	431	38	59	132				
	pSgR	**56**	9		26	21				
	pSgS	**4245**	88	4	133	4020				
	pRgNA	**539**					485	54		
	pSgNA	**294**					7	287		
	pNAgR	**675**							628	47
	pNAgS	**750**							323	427
Isoniazid Resistant	RrHr%		97.6%	96.3%	76.7%		98.6%		66.0%	
	RsHr%					10.3%		15.8%		9.9%
	95%CI		97.0–98.0	90.8–99.0	73.0–80.0	9.5–11.3	97.1–99.4	12.1–20.2	62.9–69.0	7.4–13.0

p-phenotypic, g-genotypic, DST-drug susceptibility testing, S-sensitive, R-Resistant, NA-not available.

#### Isoniazid resistance in rifampicin sensitive TB

**Fig 2:**
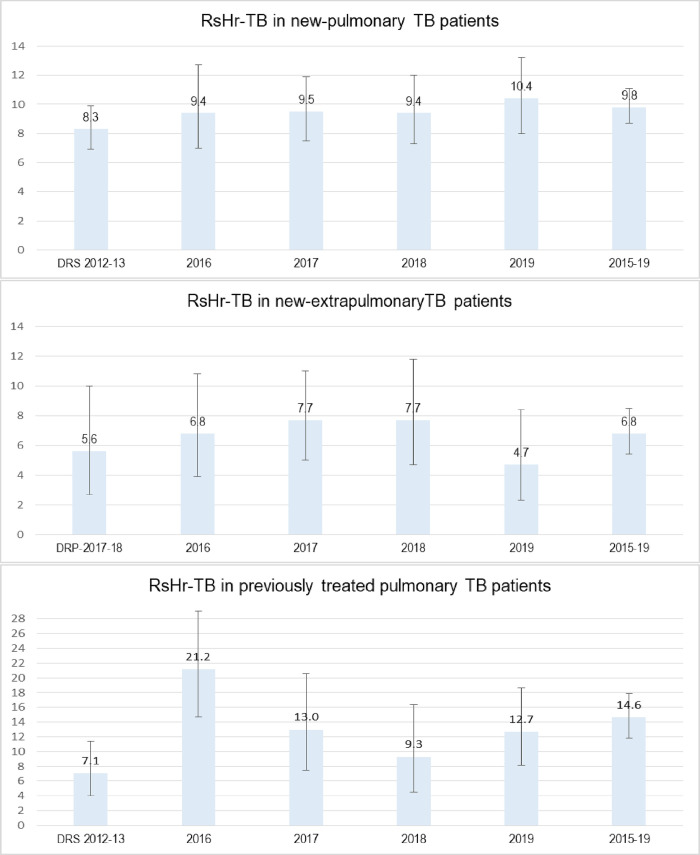
Trend of isoniazid resistance in rifampicin sensitive new pulmonary TB, new extrapulmonary TB and previously treated pulmonary TB patients, National TB reference laboratory, Pakistan, 2015–19. RsHr; Rifampicin sensitive isoniazid resistant.

Among RMP sensitive new PTB (n = 2489) and EPTB (n = 1058) patients, INH resistance (RsHr-TB) was reported in 9.8% (95%CI 8.7–11.1) and 6.8% (95%CI 5.4–8.5) respectively and 14.6% (95%11.8–17.9) among patients treated previously for new TB (n = 547) (Table 3). A stable annual trend of RsHr-TB was seen in new PTB and EPTB patients (Fig 2). A significant difference was reported in proportion of RsHr-TB between new PTB and EPTB (*P* = 0.004) and new and previously treated PTB patients *(P*<0.01). Among new PTB, significant differences were not seen between children and adults, male and females and resident of Punjab and other regions (Table 3).

**Table 3:** Isoniazid resistance among rifampicin sensitive new and previously treated pulmonary and extrapulmonary TB patients, national TB reference laboratory, Pakistan 2015–2019.

	Pulmonary TB			Extrapulmonary TB		
	New	PT*	p-value	New	PT*	p-value
**All Patients**	245/2489;9.8%	80/547;14.6%	<0.01	72/1058;6.8%	3/60;5.0%	0.588
** **	(8.7–11.1)	(11.8–17.9)		(5.4–8.5)	(1.0–13.9)	
**Gender**	** **	** **	** **	** **	** **	** **
Male	119/1263;9.4%	45/290;15.5%	0.002	28/533;5.3%	1/31;3.2%	0.608
	(7.9–11.2)	(11.5–20.2)		(3.5–7.5)	(0.08–16.7)	
Female	126/1226;10.3%	35/257;13.6%	0.122	44/525;8.4%	2/29;6.9%	0.776
** **	(8.6–12.1)	(9.7–18.4)		(6.2–11.1)	(0.8–22.8)	
**Age Group**	** **	** **	** **	** **	** **	** **
Children(<15yrs)	36/302:11.9%	7/31:22.6%	0.09	11/184:6.0%	0/6:0.0%	0.536
	(8.5–16.1)	(10.0–41.1)		(3.0–10.4)	(0.0–45.9)	
Adult	204/2147:9.5%	72/508:14.2%	0.002	61/869:7.0%	3/54:5.6%	0.694
	(8.3–10.8)	(11.3–17.5)		(5.4–8.9)	(1.1–15.4)	
Age NA	5/40:12.5%	1/8:12.5%		0/5:0.0%	0/0	
	(4.2–26.8)	(0.03–52.7)		0.00%	0.00%	
**Place of Residence**	** **	** **	** **	** **	** **	** **
Punjab province	211/2041;10.3%	64/446;14.3%	0.015	61/871;7.0%	3/48;6.3%	0.853
	(9.1–11.7)	(11.2–18.0)		(5.4–8.9)	(1.3–17.2)	
Outside Punjab	34/448;7.6%	16/101;15.8%	0.01	11/187;5.9%	0/12;0.0%	0.387
** **	(5.3–10.4)	(9.3–24.4)		(3.0–10.3)		
**AFB smear Results**	** **	** **	** **	** **	** **	** **
Positive	95/903;10.5%	65/385;16.9%	0.001	8/128;6.3%	0/17;0.0%	0.287
	(8.7–12.7)	(13.3–21.0)		(2.7–11.9)	(0.0–19.5)	
Negative	41/516;7.9%	7/86;8.1%	0.949	19/303;6.3%	2/34;5.9%	0.927
	(5.9–10.6)	(3.3–16.1)		(3.8–9.6)	(0.7–19.7)	
Not available	109/1070;10.2%	8/76;10.5%	0.934	45/627;7.2%	1/9;11.1%	
** **	(8.4–12.2)	(4.7–19.7)		(5.3–9.5)	(0.2–48.2)	

Number shown are number isoniazid resistant/ number rifampicin susceptible isolates tested, % isoniazid resistant (95%CI)

*PT; previously treated with treatment regimen recommended for new TB patients

### Molecular characterization of isoniazid resistant isolates

Among all RrHr-TB isolates, INH conferring mutations were detected in 87.1% (95%CI: 86.0–88.1) compared to 71.6% (95%CI: 67.2-75-6) in RsHr-TB (*P* < .001). A significant difference (P < .001) was also seen in frequency distribution for both *katG* (76.1% vs 41.2%) and *inhA* (7.6% and 30,2%) mutations between RrHr-TB and RsHr-TB respectively (Table 4).

**Table 4:** Molecular characterization of isoniazid resistance in rifampicin resistant and sensitive Mtb isolates from pulmonary and extrapulmonary TB patients, National TB reference laboratory Pakistan 2015–19.

	All-TB		RrHr-TB		RsHr-TB		RrHr-TB				RsHr-TB			
							PTB		EPTB		PTB		EPTB	
INH resistant isolates	N = 4542		N = 4078		N = 464		N = 3971		N = 107		N = 389		N = 75	
gWTpNWT	660	14.5%	528	12.9%	132	28.4%	519	13.1%	9	8.4%	115	29.6%	17	22.7%
Any Mutation	3882	85.5%	3550	87.1%	332	71.6%	3452	86.9%	98	91.6%	274	70.4%	58	77.3%
*katG_S315T*	3213	70.7%	3028	74.3%	185	39.9%	2943	74.1%	85	79.4%	153	39.3%	32	42.7%
*katG_315*	90	2.0%	84	2.1%	6	1.3%	84	2.1%		0.0%	5	1.3%	1	1.3%
*inhA_c-15t*	411	9.0%	287	7.0%	124	26.7%	277	7.0%	10	9.3%	101	26.0%	23	30.7%
*inhA_-15*	17	0.4%	8	0.2%	9	1.9%	8	0.2%		0.0%	9	2.3%		0.0%
*inhA_t-8c*	10	0.2%	8	0.2%	2	0.4%	8	0.2%		0.0%	1	0.3%	1	1.3%
*inhA_-8*	5	0.1%	3	0.1%	2	0.4%	2	0.1%	1	0.9%	2	0.5%		0.0%
*inhA_t-8a*	6	0.1%	3	0.1%	3	0.6%	3	0.1%		0.0%	2	0.5%	1	1.3%
*inhA_-15*,*-8*	1	0.0%	1	0.0%	0	0.0%	1	0.0%		0.0%		0.0%		0.0%
*inhA_c-15t & t-8c*	1	0.0%	1	0.0%	0	0.0%	1	0.0%		0.0%		0.0%		0.0%
*katG_S315T inhA_c-15t*	84	1.8%	84	2.1%	0	0.0%	84	2.1%		0.0%		0.0%		0.0%
*katG_S315T inhA_t-8c*	27	0.6%	27	0.7%	0	0.0%	25	0.6%	2	1.9%		0.0%		0.0%
*katG_S315T inhA_t-8a*	4	0.1%	4	0.1%	0	0.0%	4	0.1%		0.0%		0.0%		0.0%
*katG_315 inhA_c-15t*	6	0.1%	5	0.1%	1	0.2%	5	0.1%		0.0%	1	0.3%		0.0%
*katG_S315T inhA_-15*	3	0.1%	3	0.1%	0	0.0%	3	0.1%		0.0%		0.0%		0.0%
*katG_S315T inhA_-8*	3	0.1%	3	0.1%	0	0.0%	3	0.1%		0.0%		0.0%		0.0%
*katG_S315T inhA_a-16g*	1	0.0%	1	0.0%	0	0.0%	1	0.0%		0.0%		0.0%		0.0%
**Summary**														
gWTpNWT–n %	660	14.5%	528	12.9%	132	28.4%	519	13.1%	9	8.4%	115	29.6%	17	22.7%
(95%CI)	(13.5–15.6)		(11.9–14.0)		(24.4–32.8)		(12.0–14.2)		( 3.9–15.4)		( 25.1–34.4)		(13.8–33.8)	
p-value			<0.01				0.153				0.225			
*katG mutation-*n %	3303	72.7%	3112	76.3%	191	41.2%	3027	76.2%	85	79.4%	158	40.6%	33	44.0%
(95%CI)	(71.4–74.0)		(75.0–77.6)		(36.6–45.8)		(74.9–77.5)		(70.5–86.6)		(35.7–45.7)		(35.5–55.9)	
p-value			<0.01				0.443				0.584			
*inhA* mutation- n %	451	9.9%	311	7.6%	140	30.2%	300	7.6%	11	10.3%	115	29.6%	25	33.3%
95%CI	(9.1–10.8)		(6.8–8.5)		(26.0–34.4)		(6.8–8.4)		(5.2–17.7)		(25.1–34.3)		(22.9–45.2)	
p-Value			<0.01				0.300				0.523			
Double Mutation- n %	128	2.8%	127	3.1%	1	0.2%	125	3.1%	2	1.9%	1	0.3%	0	0.0%
95%CI	(2.4–3.3)		(2.6–3.7)		(0.0–1.2)		(2.6–3.7)		(0.2–6.6)		( 0.007–1.4)		(0.00–46.9)	
p-value			0.187				0.478				0.129			

RrHr-Rifampicin resistant Isoniazid resistant, RsHr-Rifampicin sensitive Isoniazid resistant

#### Rifampicin resistant TB

**Fig 3:**
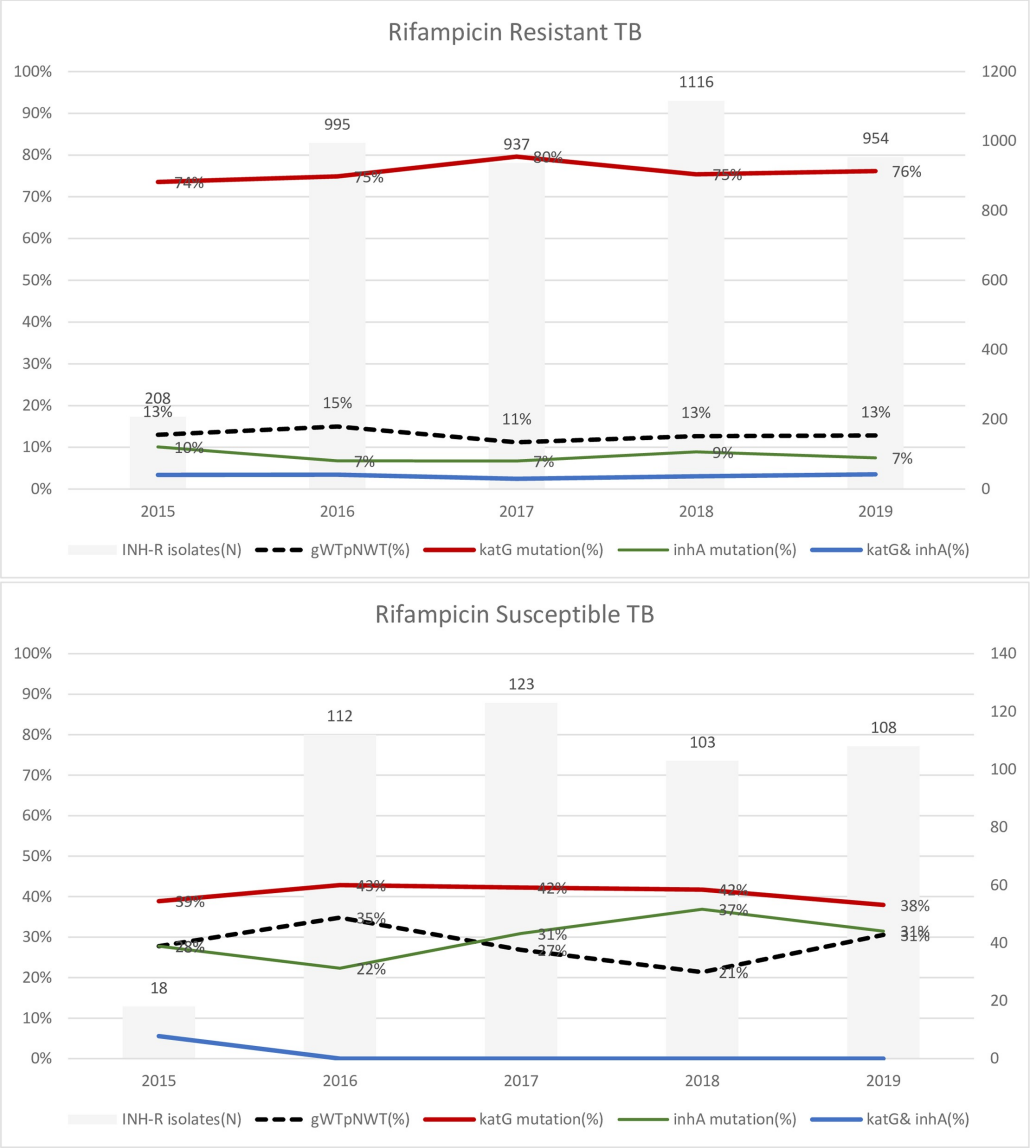
Trend of isoniazid resistance profiles associated with rifampicin resistant and rifampicin sensitive isolates, National TB reference laboratory, Pakistan 2015–19.

Among 3971 RrHr-PTB isolates, 13.1% (519) were wild type (WT) on LPA. Genetic mutations associated with INH resistance were detected in *inhA* in 7.6% and *katG* in 76.1%, and combined *katG* and *inhA* mutations in 3.1%. Among 107 RrHr-EPTB isolates, 8.4% (9) were genotypic WT, mutations were detected in *inhA* in 10.3%, *katG* in 79.4%, and double mutation in 2 isolates (1.9%) only. A significant difference was not seen between PTB and EPTB (Table 4), new and previously treated (S5 Table) and annual trends (Fig 3; S6 Table).

Among all MDR isolates (PTB and EPTB), the most frequent mutation were reported in codon S315T1 (97.2%, 3028/3112) in *katG* gene and *C-15T* in *InhA* promoter region (92.3%, 287/311) (Table 4).

#### Rifampicin sensitive TB

Among 389 RsHr-PTB isolates, 29.6% (115) were genotypic WT, INH conferring mutations were detected in *inhA in* 29.6%, *katG* gene in 40.6% and double mutation in only one isolate. Among 75 RsHr-EPTB isolates, 22.7% (17) were WT on LPA, INH conferring mutations were detected in *inhA* in 33.3%, *katG* in 44%, with no double mutation. Significant differences were not noted between isolates from PTB and EPTB (Table 4), new and previously treated (S5 Table) and annual trends. A more stable annual trend was seen for *katG* compared to *inhA* mutations. (Fig 3; S6 Table).

### Associated levofloxacin and pyrazinamide resistance

Among RMP resistant PTB isolates, resistance to LFX and PZA was reported respectively in 47.7% (95%CI: 46.2–49.2) and 44.8% (95%CI: 43.2–46.4) compared to 14.4% (13.3–15.7) and 3.7%, (95%CI: 3.0–4.5) in RMP sensitive. Similar pattern was seen in EPTB (Table 5; S7 Table).

**Table 5:** Levofloxacin and pyrazinamide resistance associated with isoniazid resistance in rifampicin resistant and sensitive *Mycobacterium Tuberculosis* isolates from pulmonary and extrapulmonary Tuberculosis patients, National TB reference laboratory 2015–2019.

Isoniazid resistance profile	Rifampicin Resistant TB					Rifampicin Sensitive TB				
	Pulmonary TB		Extrapulmonary TB		p-value	Pulmonary TB		Extrapulmonary TB		p-value
	n/N	%(95%CI)	n/N	%(95%CI)		n/N	%(95%CI)	n/N	%(95%CI)	
**Levofloxacin Resistance**										
All Isolates	1997/4183	47.7(46.2–49.2)	53/118	44.9(35.7–54.3)	0.548	486/3365	14.4(13.3–15.7)	80/1117	7.2 (5.7–8.8)	**<0.001**
H sensitive (gWTpWT)	51/214	23.8(18.3–30.1)	1/11	9.1(0.2–41.3)	0.259	388/2976	13.0(11.8–14.3)	75/1042	7.2 (5.7–8.9)	**<0.001**
H Resistant-All	1946/3969	49.0(47.5–50.6)	52/107	48.6(38.8–58.5)	0.935	98/389	25.2(21.0–29.8)	5/75	6.7(2.3–14.9)	**<0.001**
▪ gWTpNWT	213/518	41.1(36.8–45.4)	5/9	55.6 (21.2–86.3)	0.381	38/115	33.0(24.6–42.4)	1/17	5.9(0.01–28.7)	0.002
▪ *inhA* mutation	138/300	46.0(40.3–51.8)	2/11	18.2(2.3–51.8)	0.069	24/115	20.9(13.9–29.4)	1/25	4.0(0.1–20.4)	0.046
▪ *katG* mutation	1504/3026	49.7(47.9–51.5)	44/85	51.8(40.7–62.7)	0.703	36/158	22.8(16.5–30.1)	3/33	9.1(1.9–24.3)	0.076
▪ **Double Mutation**	**91/125**	**72.8(64.1–80.4)**	**1/2**	**50.0 (1.2–98.7)**	0.474	0/1	0.0(0.0–97.5)	0/0		NA
**Pyrazinamide Resistance**										
All Isolates	1655/3696	44.8(43.2–46.4)	58/104	55.8(45.7–65.5)	0.026	114/3048	3.7(3.0–4.5)	29/1019	2.8 (1.9–4.1)	0.174
H sensitive (gWTpWT)	13/202	6.4(3.5–10.8)	0/10	0.0 (0.0–30.8)	0.406	72/2704	2.7 (2.1–3.3)	23/951	2.4 (1.5–3.6)	0.618
H Resistant- All	1642/3494	47.0(45.3–48.7)	58/94	61.7(51.1–71.5)	0.005	42/344	12.2(8.9–16.1)	6/68	8.8 (3.3–18.2)	0.424
▪ gWTpNWT	133/445	29.9(25.7–34.4)	2/6	33.3(4.3–77.7)	0.857	16/99	16.2(9.5–24.9)	1/15	6.7 (0.2–31.9)	0.336
▪ *inhA* mutation	84/266	31.6(26.0–37.5)	4/10	40.0(12.2–73.8)	0.576	5/105	4.8(1.6–10.8)	5/24	20.8(7.1–42.2)	0.008
▪ *katG* mutation	**1354/2676**	**50.6(48.7–52.5)**	51/76	67.1 (55.4–77.5)	0.005	21/139	15.1(9.6–22.1)	0/29	0.0 (0.0)	0.025
▪ **Double Mutation**	**71/107**	**66.4(56.6–75.2)**	1/2	50.0(1.3–98.7)	0.672	0/1	0.0(0.00–97.5)	0/0		NA
**Combined Levofloxacin and Pyrazinamide Resistance**										
All Isolates	981/3696	26.5(25.1–28.0)	31/104	29.8(21.2–39.6)	0.453	31/3047	1.0(0.6–1.4)	2/1018	0.2(0.03–0.80)	0.013
H sensitive (gWTpWT)	6/202	3.0(0.01–6.4)	0/10	0.0(0.0–30.8)	0.578	9/2703	0.3(0.2–0.6)	2/950	0.2(0.02–0.8)	**<0.001**
H Resistant- All	975/3494	27.9(26.4–29.4)	31/94	33.0(23.6–43.4)	0.277	22/344	6.4(4.1–1.0)	0/68	0.0(0.0)	0.032
▪ gWTpNWT	56/445	12.6(9.6–16.3)	1/6	16.7 (0.4–64.1)	0.764	10/99	10.1(5.0–17.8)	0/15	0.0 (0.0)	0.198
▪ *inhA* mutation	55/266	20.7(16.0–26.0)	0/10	0.0(0.0–30.8)	0.108	1/105	1.0(0.02–5.1)	0/24	0.0(0.0)	0.623
▪ *katG* mutation	**805/2676**	**30.1(28.3–31.9)**	29/76	38.2(27.2–50.0)	0.130	11/139	7.9(4.3–13.7)	0/29	0.0 (0.0)	0.117
▪ **Double Mutation**	**59/107**	**55.1(45.2–64.8)**	1/2	50.0(12.6–98.7)	0.886	0/1	0.0(0.0–97.5)	0/0		NA

n-number of clinical isolates resistant to Levofloxacin and/or Pyrazinamide, N-Number of Isolates tested, WT-wild type, g-genotypic, p-phenotypic

#### Rifampicin resistant TB

Among RrHr-PTB isolates(n = 3969), resistance to LFX, PZA and combined LFX and PZA was 49%, 47% and 27.9%, which was higher compared to reported resistance in RrHs-TB (n = 214) of 23.8%, 6.4% and 3.0%. Similarly among RrHr-EPTB (n = 107) reported resistance of 48.6%, 61.7% and 33.0% was higher compared to 9.1%, 0% and 0% in RrHs-TB (n = 11) (Table 5).Within RrHr-TB, significant difference in resistance was not seen between PTB and EPTB (*P* = .08) (Table 5), new and previously treated TB (*P =* .956) (S7 Table) and annual trends (S8 Table).

#### Rifampicin sensitive TB

Among RsHr-PTB(n = 389), resistance to LFX, PZA and combined LFX and PZA, isolates was reported in 25.2%, 12.2% and 6.4% respectively compared to 13.0%, 3.7% and 1.0 in RsHs-PTB(n = 2976). Among RsHr-EPTB (N = 75)isolates, resistant was reported respectively in 6.7%, 8.8% and 0% compared to 7.2%, 2.8% and 0.2% in RsHs-EPTB (N = 1042).No significant changes were see in the annual trend of resistance to LFX and PZA. (S8 Table).

#### Isoniazid conferring mutations and Levofloxacin and pyrazinamide resistance

Among RrHr-PTB isolates, a relative higher resistance to LFX was seen in isolates with double mutations and relative higher resistance to PZA and combined LFX and PZA resistance was seen associated with *katG* and double mutations. (Table 5; S7 Table; S1 Fig).

## Discussion

We analyzed a large DST data set from 11045 patients registered during 2015–2019 in NTRL, Pakistan. During the study period, pDST was performed on 87%, gDST on 92% and 79.5% of isolates were tested by both methods. We analyzed results of 8787 Mtb isolates including 1236 from EPTB patients, tested by both phenotypic and genotypic methods. Key findings of our analysis include significant difference in resistance detected by WHO recommended pDST (MGIT 960) and gDST (LPA) methods for both RMP and INH, a significantly lower sensitivity of gDST to detect INH resistance in RMP sensitive TB, difference in genetic profiles of INH resistance associated with RMP resistant and sensitive TB, a higher level of LFX and PZA resistance associated with INH resistance in RMP resistant and sensitive population. Additionally we noted a stable annual trend of INH resistance in RMP sensitive new TB patients, INH resistance profiles, associated FQ and PZA resistance and INH resistance missed by gDST method.

We reported, an INH resistance of 9.8% (95%CI; 8.7–11.1) in new and 14.6% (95%CI: 11.8–17.9) in previously treated RMP sensitive PTB patients. Among new PTB, a higher point estimates compared to the estimates of DRS (8.3%, 95%CI: 7.0–10.7) conducted in 2013 [14] can be explained based on the possibility of a selected higher risk individuals among new RMP sensitive patients referred for testing in routine settings. However a stable RsHr resistance trend was seen during the study period with an insignificant increase only in 2019. Among previously treated, compared to DRS, the proportion of RsHr-TB was significantly higher but fluctuations were seen in annual trends. Fluctuation in resistance was most likely due to variation in proportion of relapse vs failures of previous treatment among patient tested. However, a higher RsHr reported among previously treated was consistent with findings of other similar studies from Pakistan [20–22]. Among children with PTB (n = 302), estimated RsHr-TB of 11.9%(8.5–16.1), was higher but not statistically different from adults(p = .189) and was consistent with the global estimates of 12.1% (95%CI:9.8% to 14.8%) among all childhood TB cases [23]. However prevalence estimates in children can also be argued as not being a true representative of childhood TB population as most likely specimens from sicker children having access to specialized TB care and diagnostic facilities were tested. Among RMP sensitive new EPTB patient (n = 1058), INH resistance of 6.8% (95%CI; 5.4–8.5) was significantly lower compared to PTB (*P* < .001) in same study population but was consistent with primary drug resistance (5.6%, 95CI; 2.7–10.0) reported in EPTB [19] and PTB in Pakistan [14, 20, 24]. Most of the EPTB patients in contrast to PTB, were referred for diagnosis of TB and only a few had history of previous TB treatment (<10%), with possibility of these estimates being a true reflection of RsHr in EPTB at population level.

Among Mtb isolates tested by both methods (n = 8787), discordance in RMP results was reported in 7.7% of all and 15.8% (678/4303) of RMP resistant isolates. Discordance in RMP results was also reported previously in random population-based DRS (2013) in which all samples were tested in parallel using Xpert MTB/Rif and Lowenstein Jensen (LJ) media and sequencing was performed to confirm and resolve discordance in RMP results [14]. However in DRS, discordance of 11.7% was reported among all RMP resistant isolates, including 4% (4/104) missed on pDST and 7.7% (8/104) by gDST. Contrary to DRS, in this study 13.2% (n = 570) were missed by MGIT (pDST) and 2.5% (n = 108) by LPA (gDST). The most plausible explanation for a higher number of observed missed RMP resistance by pDST (13.2% vs 4%) in study sample was due to use of automated liquid DST (MGIT), which is known to miss higher proportion of RMP resistant cases compared to LJ media [25, 26]. On the other hand the lower proportion of RMP resistant cases missed by gDST (7.7% vs 2.5%) was most likely an effect of the current diagnostic algorithm followed in programme settings in which patients reported RMP sensitive are initiated on standard first line treatment and are not investigated further unless they fail to respond or fail treatment.

Recently conducted systematic review and meta-analysis, reported significant higher failure rate among INH resistant compared to susceptible TB patients when treated with standard first-line drugs regimens [13]. Limitations of currently available pDST and gDST in detecting RMP resistance are well recognized [25–27]. Selection of either one of the two DST method for routine practice is likely to result in important diagnostic and clinical implications. In our study population, without genotypic DST, 10.1% (411/4078) of the MDR would have been reported as RsHr-TB and treatment in these instances with standard first line treatment regimen would likely have resulted in suboptimal treatment outcomes. In a recently published study, a non-negligible extent of misclassifying MDR-TB as INH-resistant TB is demonstrated and impact of treating patients with missed RMP resistance for RsHr-TB with WHO recommended FQ containing regimen is strongly argued [27]. In another study from South Africa 15% of INH resistant isolates initially tested negative for RMP resistance by all three WHO-endorsed commercial tests were reclassified as MDR on identification of resistance conferring mutation (*rpoB* Ile491phe) using deep sequencing [28].

In our data set, contrary to RMP, a higher proportion of INH resistance (14.5%; n = 660) was missed by gDST. Our finding are consistent with the results of a recent study from eastern DRC, in which INH resistance was reported in only 55% of the RMP resistant cases detected by Xpert MTB/RIF on subsequent testing by LPA, raising an argument on use of RMP resistance as surrogate marker for MDR [29].

We studied 4542 INH resistant isolates for molecular markers and mutations causing INH resistance were identified in 85.5% with frequency of *katG*, *inhA* and combined *katG and inhA* mutations in 72.7%, 9.9% and 2.8% respectively. Our findings are consistent with the published data [11, 12, 30] but with a lower proportion of combined *katG* and *inhA* mutations in our population [12, 31]. INH resistance profiles when studied, stratified by RMP results, significant differences were reported between RrHr-TB (n = 4078) and RsHr-TB(n = 464) with regard to proportion of INH conferring mutation detected (87.1% vs 71.6%) and frequency of the mutations in *inhA* (7.6 vs 30.2%), *katG* (76.3 vs 41.2%) and combined *inhA* and *katG* (3.1% vs 0.2%). The distribution of INH resistance mutations among RsHr-TB is less well mapped globally, however our findings are consistent with the estimates from an international collection of over 5,000 strains with reported frequency of S315T *kat*G in 88.7% of MDR and 61.3% of RsHr-TB showing substantially higher representation of *katG* among MDR strains [32].

In our study population of RsHr-TB, 41% had mutation in *katG*, 30% in *inhA* and only one isolates had combined mutation. Published data on the influence of genotype on treatment outcomes of RsHr-TB are conflicting, study from Vietnam, suggest that *katG* mutations and not *inhA* are associated with unfavorable treatment outcomes [33]. Whereas in a study from South Africa, no evidence was found suggesting that specific isoniazid resistance conferring mutations are associated with poor treatment outcomes, and results showed that patients with *katG* mutations had greater odds of successful outcome when treated with high-dose isoniazid compared to those who received standard dose [34].Few studies have also evaluated the effectiveness of high-dose isoniazid in patients with DRTB and limited data available suggest clinical benefit without a higher toxicity [35, 36]. A recent study has demonstrated that high dose INH in MDR patients with *inhA* mutations has similar magnitude of bactericidal activity as with standard doses in drug-susceptible TB patients [37].

In Pakistan, one of the key challenge for treating MDR-TB and RsHr-TB is high FQ resistance. We reported a higher FQ resistance in RMP resistant and sensitive isolates compared to population level resistance [12, 15] but was consistent with previous laboratory based study from Pakistan [38]. In RMP resistant and sensitive population, significantly higher resistance to LFX and PZA was noted associated with INH resistance and was statistically higher for all INH resistance profiles. However a relative higher resistance was seen associated with combined *katG* and *inhA* mutations, consistent with findings of a recent study [31]. A high LFX resistance and lower frequency of *katG* mutations in RsHr-TB in our study population imply consideration for high dose INH rather than LFX as a treatment option.

The End TB Strategy released in 2015 calls for the early diagnosis of TB including universal DST [39]. Xpert MTB/RIF assay was endorsed by WHO in 2010, however even in 2018, among bacteriologically confirmed TB patients, only 51% globally and 45% in Pakistan were tested for RMP resistance [7]. Diagnostic and operational challenges to implement universal INH testing and treatment for INH resistant TB, are more complex, because of the larger population of RMP sensitive patients and complex laboratory capacity required for testing in the absence of a rapid and convenient diagnostic platform for detection of INH resistance and challenges faced in decentralization of LPA in most of HBC settings. Additionally in Pakistan country context, even with systematic testing, 30 percent of the INH resistance are likely to be missed if tested by LPA only, second, there is lack of laboratory capacity to systematically exclude RMP resistance which are missed by Xpert MTB/Rif, third, high LFX resistance makes FQ testing mandatory for all and lastly, FQ resistance in a substantial number of RsHr-TB patients will render them ineligible for recommended treatment.

Currently HBC are struggling to develop capacity for comprehensive second line DST including new and repurpose drugs [5] and to establish sequencing capacity to diagnose RMP and other drug resistance not detected by routine tests. Plans to implement universal testing and treatment for INH resistant TB with existing capacity are likely to overwhelm laboratory systems at the cost of compromising services for RMP resistant TB patients. A new GeneXpert cartridge for testing INH and FQ resistance is expected to be available in near future [40, 41], however countries will need to invest in procurement of next generation modules and to make it available at same level of health care as RMP testing for detection of INH and FQ resistance in RMP sensitive TB patients in parallel with plans to implement treatment for INH resistance. In addition performance evaluation of new diagnostic tests in both RMP resistant and sensitive population in different geographical settings also needs consideration.

The value of any such laboratory study would be greatly increased if treatment outcomes could be linked to the resistance profile. Studies are also needed to fill knowledge gap in isoniazid acetylator status of the population in HBC and to evaluate effectiveness, optimal dosing and potential toxicity of high-dose isoniazid to offer simple treatment options applicable in program settings.

We studied retrospective data and possibility of errors in routinely collected patient information cannot be excluded. Furthermore findings of this study cannot be generalized to the population level. However laboratory based surveillance can complement random survey as analysis provides information on drug resistance pattern in a large dataset of DRTB patients at time of treatment initiation and can guide in country specific planning for TB diagnostics, diagnostic algorithm and appropriate treatment regimens for drug resistance in TB patients.

## Supporting information

S1 Table Demographic and clinical characteristics of pulmonary and extrapulmonary tuberculosis patients tested for drug susceptibility at national TB reference laboratory Pakistan, 2015–2019. (PDF)

S2 Table Specimen types from pulmonary and extrapulmonary tuberculosis patients processed for drug susceptibility testing, national TB reference laboratory Pakistan, 2015–19. (PDF)

S3 Table Annual trend and difference in isoniazid and rifampicin resistance detected by phenotypic and genotypic DST methods, in pulmonary and extrapulmonary tuberculosis patients, national TB reference laboratory Pakistan 2015–19. R-rifampicin, H-isoniazid, r-resistant, s-susceptible, DST-drug susceptibility testing, p-phenotypic; g-genotypic.
(PDF)

S4 Table Rifampicin and isoniazid resistance trend and correlation between phenotypic and genotypic drug susceptibility testing, national TB reference Laboratory, Pakistan 2015–2019. RMP-rifampicin, INH-isoniazid, R-resistant, S-sensitive, pDST-phenotypic, drug susceptibility testing; gDST-Genotypic drug susceptibility testing.
(PDF)

S5 Table Molecular characterization of isoniazid resistance in Mtb isolates from pulmonary and extrapulmonary TB patients, stratified by rifampicin resistance and history of TB treatment, national TB reference laboratory, Pakistan 2015–2019. INH-isoniazid.
(PDF)

S6 Table Annual trend of genotypic profiles of isoniazid resistance associated with rifampicin resistant and sensitive Mtb isolates from new and previously treated TB patients, national TB reference laboratory, Pakistan, 2015–19. RMP-rifampicin, INH-isoniazid, PT-Previously treated.
(PDF)

S7 Table Levofloxacin and pyrazinamide resistance associated with isoniazid resistance in rifampicin resistant and sensitive Mtb isolates from pulmonary and extra pulmonary TB patient, stratified by history of TB treatment, national TB reference laboratory Pakistan, 2015–19. n-Number of isolates resistant to Levofloxacin and/or Pyrazinamide, N- Number of isolates tested INH-isoniazid.
(PDF)

S8 Table Annual trend of levofloxacin and pyrazinamide resistance associated with isoniazid resistance in rifampicin resistant and sensitive Mtb isolates from pulmonary and extrapulmonary TB patients, national TB reference laboratory Pakistan, 2015–19. LFX-Levofloxacin, PZA-Pyrazinamide, n = Number of isolates resistant, N = Number of isolates tested.
(PDF)

S1 Fig Levofloxacin, pyrazinamide and combined levofloxacin and pyrazinamide resistance associated with isoniazid sensitive, isoniazid resistant and specific isoniazid conferring mutations in rifampicin resistant and sensitive isolates from pulmonary and extrapulmonary TB patients. National TB reference Laboratory, Pakistan 2015–2019. Rr-Rifampicin resistant, Rs-Rifampicin sensitive, Hr-Isoniazid resistance, Hs-Isoniazid sensitive, isoniazid sensitive.
(TIF)

## References

[1] Mitchison DA. Role of individual drugs in the chemotherapy of tuberculosis. International Journal of Tuberculosis and Lung Disease.2000; 4(9):796–806. 10985648

[2] Stagg HR, Lipman MC, Mchugh TD, Jenkins HE. Isoniazid-resistant tuberculosis: a cause for concern?. International Journal of Tuberculosis and Lung Disease. 2017;129–139. 10.5588/ijtld.16.0716 28234075 PMC5479083

[3] Van Deun A, Decroo T, Piubello A, Jong BC, De Lynen L, Rieder HL. Principles for constructing a tuberculosis treatment regimen: the role and definition of core and companion drugs. International Journal of Tuberculosis and Lung Disease. 2018; 239–245. 10.5588/ijtld.17.0660 29471899

[4] World Health Organization. Guidelines for treatment of drug-susceptible tuberculsois and patient care. 2017 Available:http://www.who.int/tb/publications/2017/dstb_guidance_2017/e

[5] World Health Organization. WHO consolidated guidelines on drug-resistant tuberculosis treatment. 2019. 30946559

[6] World Health Organization. Latent tuberculosis infection—updated and consolidated guidelines for pro- grammatic management. 2018 Available: https://www.who.int/tb/publications/2018/executivesummary_consolidated_guidelines_ltbi.pdf?ua= 30277688

[7] World Health Organization. Global Tuberculosis Report. 2019 Available: https://apps.who.int/iris/bitstream/handle/10665/329368/9789241565714-eng.pdf?ua=1

[8] Lempens P, Meehan CJ, Vandelannoote K, Fissette K, De Rijk P, Van Deun A, et al Isoniazid resistance levels of Mycobacterium tuberculosis can largely be predicted by high-confidence resistance-conferring mutations. Scientific Reports. 2018; 8(1), 1–9. 10.1038/s41598-017-17765-5 29459669 PMC5818527

[9] Ghodousi A, Tagliani E, Karunaratne E, Niemann S, Perera J, Köser CU, et al Isoniazid resistance in mycobacterium tuberculosis is a heterogeneous phenotype composed of overlapping mic distributions with different underlying resistance mechanisms. Antimicrobial Agents and Chemotherapy. 2019; 63(7), 17–20. 10.1128/AAC.00092-19 PMC6591585 31010866

[10] Dantes R, Metcalfe J, Kim E, Kato-Maeda M, Hopewell PC, Kawamura M, et al Impact of isoniazid resistance-conferring mutations on the clinical presentation of isoniazid monoresistant tuberculosis. PLoS ONE. 2012; 7(5), 3–7. 10.1371/journal.pone.0037956 PMC3359338 22649569

[11] Seifert M, Catanzaro D, Catanzaro A, Rodwell TC. Genetic mutations associated with isoniazid resistance in Mycobacterium tuberculosis: a systematic review. PloS ONE. 2015; 10, e0119628, 10.1371/journal.pone.0119628 25799046 PMC4370653

[12] Dean AS, Zignol M, Cabibbe AM, Falzon D, Glaziou P, Cirillo DM, et al Prevalence and genetic profiles of isoniazid resistance in tuberculosis patients: A multicountry analysis of cross-sectional data. PLoS Medicine. 2020;17(1), e1003008 10.1371/journal.pmed.1003008 PMC6974034 31961877

[13] Gegi M, Winters N, Benedetti A, Soolingen D Van, Menzies D.Treatment of isoniazid-resistant tuberculosis with first-line drugs: a systematic review and meta-analysis. The Lancet Infectious Diseases. 2017; 17(2), 223–234. 10.1016/S1473-3099(16)30407-8 27865891

[14] Tahseen S, Qadeer E, Khanzada FM, Rizvi AH, Dean A, Van Deun A et al Use of Xpert® MTB/RIF assay in the first national antituberculosis drug resistance survey in Pakistan. International Journal of Tuberculosis and Lung Disease. 2016; 20(4). 10.5588/ijtld.15.0645 26970152

[15] Zignol M, Dean AS, Alikhanova N, Andres S, Cabibbe AM, Cirillo DM, et al Population-based resistance of Mycobacterium tuberculosis isolates to pyrazinamide and fluoroquinolones: results from a multicountry surveillance project. The Lancet Infectious Diseases. 2016;16(10). 10.1016/S1473-3099(16)30190-6 PMC5030278 27397590

[16] Technical Report on critical concentrations for drug susceptibility testing of medicines used in the treatment of drug-resistant tuberculosis. Geneva: World Health Organization; 2018 (WHO/CDS/TB/2018.5). License: CC BY-NC-SA 3.0 IGO

[17] Global Laboratory inititiative(GLI), StopTB partnership, Mycobacteriology laboratory manual 1st edition. 2014; http://www.who.int/tb/laboratory/mycobacteriology-laboratory-manual.pdf accessed on 1st July 2020.

[18] Global Laboratory inititiative(GLI), StopTB partnership, Line probe assays for drug resistant tuberculosis detection Interpretation and reporting guide for laboratory staff and clinicians http://www.stoptb.org/wg/gli/assets/documents/LPA_test_web_ready.pdf, accessed on 1st July 2020.

[19] Tahseen S, Ambreen A, Masood F, Qadir M, Hussain A, Jamil M et al Primary drug resistance in extra-pulmonary tuberculosis: a hospital-based prospective study from Pakistan, INT J TUBERC LUNG DIS. 2019; 23(8):900–906 Q 2019 The Union 10.5588/ijtld.18.0531 31533879

[20] Ayaz A, Hasan Z, Jafri S, Inayat R, Mangi R, Ali A, et al International Journal of Infectious Diseases Characterizing Mycobacterium tuberculosis isolates from Karachi, Pakistan: drug resistance and genotypes. International Journal of Tuberculosis and Lung Disease. 2012; 16(4), e303–e309. 10.1016/j.ijid.2011.12.015 22365136

[21] Javaid A, Hasan R, Zafar A, Chaudry MA, Qayyum S, Qadeer E, et al Pattern of first- and second-line drug resistance among pulmonary tuberculosis retreatment cases in Pakistan. International Journal of Tuberculosis and Lung Disease. 2017; 303–308. 10.5588/ijtld.16.0444 28225340

[22] Fasih N, Rafiq Y, Jabeen K, Hasan R. High Isoniazid Resistance Rates in Rifampicin Susceptible Mycobacterium tuberculosis Pulmonary Isolates from Pakistan. PLoS ONE. 2012; 7(11), 9–12. 10.1371/journal.pone.0050551 PMC3511527 23226311

[23] Yuen CM, Jenkins HE, Rodriguez CA, Keshavjee S, Becerra MC. Global and regional burden of isoniazid-resistant tuberculosis. Pediatrics, 2015; 136(1), e50–e59. 10.1542/peds.2015-0172 26034243 PMC4485010

[24] Javaid A, Hasan R, Zafar A, Ghafoor A, Pathan AJ, Rab A. et al Prevalence of primary multidrug resistance to anti-tuberculosis drugs in Pakistan. International Journal of Tuberculosis and Lung Disease. 2008; 12(3):326–331 18284840

[25] Van Deun A, Aung KJ, Bola V, Lebeke R, Hossain MA, de Rijk WB, et al Rifampin drug resistance tests for tuberculosis: challenging the gold standard. J Clin Microbiol. 2013; 51(8):2633–2640. [PubMed: 10.1128/JCM.00553-13 ]. 23761144 PMC3719626

[26] Hoffmann H, Hillemann D, Rigouts L, Deun A Van, Kranzer K. How should discordance between molecular and growth-based assays for rifampicin resistance be investigated?. International Journal of Tuberculosis and Lung Disease. 2017; 21(7):721–726. 10.5588/ijtld.17.0140 28633695

[27] Van Deun A, Decroo T, Kya Jai Maug A, Hossain MA, Gumusboga M, Mulders W, et al The perceived impact of isoniazid resistance on outcome of first-line rifampicin-throughout regimens is largely due to missed rifampicin resistance. PLoS ONE. 2020; 15(5): e0233500 10.1371/journal.pone.0233500 32421749 PMC7233532

[28] Makhado NA, Matabane E, Faccin M, Pinc¸on C, Jouet A, Boutachkourt F, et al Outbreak of multidrug- resistant tuberculosis in South Africa undetected by WHO-endorsed commercial tests: an observational study. Lancet Infect Dis. 2018; 18:1350–9. 10.1016/S1473-3099(18)30496-1 30342828

[29] Nachega JB, Theron G, Metcalfe JZ, Francisco S, Diacon A. Xpert MTB / RIF-detected Rifampicin Resistance is a Sub-Optimal Surrogate for Multidrug Resistant Tuberculosis in Eastern Democratic Republic of the Congo: Diagnostic and Clinical Implications. Clinical Infectious Diseases. 2020; 10.1093/cid/ciaa873 PMC8282324 32590841

[30] Siddiqui S, Brooks MB, Malik AA, Fuad J, Nazish A, Bano S, et al Evaluation of GenoType MTBDRplus for the detection of drug-resistant Mycobacterium tuberculosis on isolates from Karachi, Pakistan. PLoS ONE. 2019; 14(8): e0221485 10.1371/journal.pone.0221485 31425565 PMC6699735

[31] Kurbatova EV, Click ES, Alexander H, Cegielski JP, Posey JE, Ershova J, et al Isoniazid and Rifampin-Resistance Mutations Associated With Resistance to Second-Line Drugs and With Sputum Culture Conversion. JID. 2020; 221(12).2072–2082,. 10.1093/infdis/jiaa042 32002554 PMC7289556

[32] Manson AL, Cohen KA, Abeel T, Desjardins CA, Derek TA, Clifton EB III, et al Genomic analysis of globally diverse Mycobacterium Tuberculosis strains provides insights into the emergence and spread of multidrug resistance. Nat Genet. 2017; 49(3): 395–402. 10.1038/ng.3767 28092681 PMC5402762

[33] Huyen MN, Cobelens FG, Buu TN, Lan NT, Dung NH, Kremer K, et al Epidemiology of isoniazid resistance mutations and their effect on tuberculosis treatment outcomes. Antimicrobial Agents and Chemotherapy. 2013; 57(8), 3620–3627. 10.1128/AAC.00077-13 23689727 PMC3719713

[34] Jacobson KR, Theron D, Victor TC, Streicher EM, Warren RM, Murray MB. Treatment outcomes of isoniazid-resistant tuberculosis patients, Western Cape Province, South Africa. Clin Infect Dis 2011; 53:369–72. 10.1093/cid/cir406 21810750 PMC3202325

[35] Katiyar SK, Bihari S, Prakash S, Mamtani M, Kulkarni H. A randomised controlled trial of high-dose isoniazid adjuvant therapy for multidrug-resistant tuberculosis. International Journal of Tuberculosis and Lung Disease. 2008;12:139–145. 18230245

[36] Walsh KF, Vilbrun SC, Souroutzidis A, Delva S, Joissaint G, Mathurin L, et al Improved outcomes with high-dose isoniazid in multidrugresistant tuberculosis treatment in Haiti. Clin Infect Dis 2019;69: 717–719 10.1093/cid/ciz039 30698688 PMC7322692

[37] Dooley KE, Miyahara S, von Groote-Bidlingmaier F, Sun X, Hafner R, Rosenkranz SL, et al; A5312 Study Team. Early bactericidal activity of different isoniazid doses for drug resistant TB (INHindsight): a randomized open-label clinical trial. Am J Respir Crit Care Med. 2020; 10.1164/rccm.201910-1960OC 31945300 PMC7258626

[38] Jabeen K, Shakoor S, Chishti S, Ayaz A, Hasan R. Fluoroquinolone-resistant *Mycobacterium tuberculosis*, Pakistan, 2005–2009 [letter]. Emerg Infect Dis [serial on the Internet]. 2011 10.3201/eid1703.100957 PMC3166006 21392465

[39] The end TB strategy, Global strategy and targets for tuberculosis prevention, care and control after 2015. Geneva, World Health Organization, 2015(WHO/HTM/TB/2015.19). https://www.who.int/tb/End_TB_brochure.pdf?ua=1

[40] Chakravorty S, Roh SS, Glass J, Smith LE, Simmons AM, Lund K, et al Detection of isoniazid-, fluoroquinolone-, amikacin-, and kanamycin-resistant tuberculosis in an automated, multiplexed 10-color assay suitable for point-of-care use. J Clin Microbiol 2017 55:183–198. 10.1128/JCM.01771-16 27807153 PMC5228229

[41] FIND press release, New rapid molecular test for tuberculosis can simultaneously detect resistance to first- and second-line drugs 16 July 2020, Available from https://www.finddx.org/wp-content/uploads/2020/07/PR_Cepheid-2020-CID-RespiratoryXpert-MTB-XDR.pdf

