# Supplementary material for: Isoniazid resistance profile and associated levofloxacin and pyrazinamide resistance in rifampicin resistant and sensitive isolates/from pulmonary and extrapulmonary tuberculosis patients in Pakistan: A laboratory based surveillance study 2015-19
Source: PLoS One. 2020 Sep 23;15(9):e0239328. doi: 10.1371/journal.pone.0239328 (PMC7511002; doi:10.1371/journal.pone.0239328)
Supplement: S1 Table — (PDF) [file pone.0239328.s001.pdf]

S1-Table: Demographic and clinical characteristics of pulmonary and extrapulmonary tuberculosis patients tested for drug susceptibility at National TB Reference laboratory, Pakistan, 2015-2019

	ALL TB N=11045		Pulmonary TB N=9647		Extrapulmonary TB N=1398	
Median age (IQR)	30(20,48)		32(22,50)		23(17,35)	
**Gender**						
Male	5660	51.2%	4957	51.4%	703	50.3%
Female	5385	48.8%	4690	48.6%	695	49.7%
**Age Group**						
0-14	831	7.5%	592	6.1%	239	17.1%
15-24	3094	28.0%	2586	26.8%	508	36.3%
25-34	2167	19.6%	1889	19.6%	278	19.9%
35-44	1451	13.1%	1305	13.5%	146	10.4%
45-54	1419	12.8%	1337	13.9%	82	5.9%
55-64	1057	9.6%	984	10.2%	73	5.2%
>65	834	7.6%	767	8.0%	67	4.8%
Not Available	192	1.7%	187	1.9%	5	0.4%
**Place of residence**						
Punjab	9502	86.0%	8340	86.5%	1162	83.1%
Khyber Pakhtunkhwa	328	3.0%	328	3.4%		0.0%
Balochistan	9	0.1%	9	0.1%		0.0%
Islamabad capital territory	1086	9.8%	853	8.8%	233	16.7%
Gilgit Baltistan	20	0.2%	20	0.2%		0.0%
Azad Jammu Kashmir	100	0.9%	97	1.0%	3	0.2%
**AFB Smear Results**						
Positive	6188	56.0%	5984	62%	204	14.6%
Negative	1918	17.4%	1533	16%	385	27.5%
Not available	2939	26.6%	2130	22%	809	57.9%
**History of previous TB treatment**						
No	5526	50.0%	4244	44.0%	1282	91.7%
Yes –All	5519	50.0%	5403	56.0%	116	8.3%
• For new TB	2473	44.8%	2391	44.3%	82	70.7%
• For retreatment TB	650	11.8%	639	11.8%	11	9.5%
• For DRTB	1216	22.0%	1208	22.4%	8	6.9%
• Yes-Other	815	14.8%	808	15.0%	7	6.0%
• Not available	365	6.6%	357	6.6%	8	6.9%
**Referral by Health care tier**						
Tertiary health care	7273	65.8%	6132	64.0%	1141	81.6%
Secondary health care	3334	30.2%	3111	32.0%	223	16.0%
Primary health care	438	3.9%	404	4.0%	34	2.4%
**Referral by health care TB services**						
Drug resistant TB	9258	83.8%	8237	85.4%	1021	73.0%
Drug sensitive TB	1787	16.2%	1410	14.6%	377	27.0%
