# Supplementary material for: Isoniazid resistance profile and associated levofloxacin and pyrazinamide resistance in rifampicin resistant and sensitive isolates/from pulmonary and extrapulmonary tuberculosis patients in Pakistan: A laboratory based surveillance study 2015-19
Source: PLoS One. 2020 Sep 23;15(9):e0239328. doi: 10.1371/journal.pone.0239328 (PMC7511002; doi:10.1371/journal.pone.0239328)
Supplement: S2 Table — (PDF) [file pone.0239328.s002.pdf]

S2-Table: Specimen types from pulmonary and extrapulmonary tuberculosis patients processed for drug susceptibility testing, National TB reference laboratory Pakistan, 2015-19.

**All Cases**	**2015**	**2016**	**2017**	**2018**	**2019**	**2015-2019**			
	**pDST and/or gDST**					**pDST and/or gDST**		**pDST and gDST**	
**All TB Cases**	**965**	**2214**	**2615**	**2628**	**2623**	**11045**		**8787**	
**Pulmonary specimen**	**857**	**1897**	**2202**	**2333**	**2358**	**9647**		**7551**	
Sputum	816	1549	1774	2093	2125	8357	86.6%	6372	84.4%
Broncho alveolar Lavage	10	197	248	115	172	742	7.7%	673	8.9%
Tracheal Lavage		1	3	8	3	15	0.2%	14	0.2%
Gastric Lavage/Washing	31	148	176	117	56	528	5.5%	487	6.4%
Lung Biopsy		2	1		2	5	0.1%	5	0.1%
**Extra Pulmonary specimen**	**108**	**317**	**413**	**295**	**265**	**1398**		**1236**	
Lymph Node	6	128	195	141	103	573	41.0%	537	43.4%
Pleural Fluid	4	82	87	51	62	286	20.5%	274	22.2%
Cerebrospinal fluid	4	26	21	22	14	87	6.2%	83	6.7%
Pericardial Fluid		1	5	6	2	14	1.0%	13	1.1%
Urine			1	4	2	7	0.5%	5	0.4%
Other	4	11	11	6	8	40	2.9%	36	2.9%
EPTB disease site not specified(NOS)	83					83	5.9%		0.0%
Fine needle aspiration (NOS)		1	4	4	3	12	0.9%	11	0.9%
Pus (NOS)	4	57	69	49	55	234	16.7%	218	17.6%
Tissue Biopsy(NOS)	3	11	20	12	16	62	4.4%	59	4.8%

pDST-Phenotypic drug susceptibility testing ; gDST-Genotypic drug susceptibility testing
