# Supplementary material for: Isoniazid resistance profile and associated levofloxacin and pyrazinamide resistance in rifampicin resistant and sensitive isolates/from pulmonary and extrapulmonary tuberculosis patients in Pakistan: A laboratory based surveillance study 2015-19
Source: PLoS One. 2020 Sep 23;15(9):e0239328. doi: 10.1371/journal.pone.0239328 (PMC7511002; doi:10.1371/journal.pone.0239328)
Supplement: S3 Table — R-rifampicin, H-isoniazid, r-resistant, s-susceptible, DST-drug susceptibility testing, p-phenotypic; g-genotypic. (PDF) [file pone.0239328.s003.pdf]

S3-Table: Annual trend and difference in isoniazid and rifampicin resistance between phenotypic and genotypic DST methods in pulmonary and extrapulmonary tuberculosis patients, National TB reference laboratory, Pakistan 2015-19

	ALL	Pulmonary Tuberculosis						Extrapulmonary Tuberculosis					
ALL DST	Total	2015	2016	2017	2018	2019	Total	2015	2016	2017	2018	2019	Total
	11045	857	1897	2202	2333	2358	9647	108	317	413	295	265	1398
**Phenotypic DST**													
All pDST	9620	850	1764	1903	1951	1800	8268	108	313	400	286	245	1352
Any Rr-TB%<br>(95%CI)	4225;43.9%<br>(42.9-44.9)	594;69.9%<br>(66.7-73.0)	932;52.8%<br>(50.5-55.2)	854;44.9%<br>(42.6-47.1)	943;48.3%<br>(46.1-50.6)	794;44.1%<br>(41.8-46.4)	4117;49.8%<br>(48.7-50.9)	20;18.5%<br>(11.7-27.1)	25;8.0%<br>(5.2-11.6)	29;7.3%<br>(4.9-10.2)	20;7.0%<br>(4.3-10.6)	14;5.7%<br>(3.2-9.4)	108;8.0%<br>(6.6-9.6)
Any Hr-TB%<br>(95%CI)	5025;52.2%<br>(51.2-53.2)	650;76.5%<br>(73.5-79.3)	1076;61.0%<br>(58.7-63.3)	1008;53.0%<br>(50.7-55.2)	1118;57.3%<br>(55.1-59.5)	976;54.2%<br>(51.9-56.5)	4828;58.4%<br>(57.3-59.5)	27;25.0%<br>(17.2-34.3)	40;12.8%<br>(9.3-17.0)	60;15.0%<br>(11.6-18.9)	44;15.4%<br>(11.4-20.1)	26;10.6%<br>(7.1-15.20	197;14.6%<br>(12.7-16.6)
MDR/Rr-TB	4117/4225	582/594	900/932	837/854	921/943	770/794	4010/4117	20/20	24/25	29/29	20/20	14/14	107/108
MDR%<br>(95%CI)	97.4%<br>(96.9-97.9)	98.0%<br>(96.5-99.0)	96.6%<br>(95.2-97.6)	98.0%<br>(96.8-98.8)	97.7%<br>(96.5-98.8)	97.0%<br>(95.5-98.1)	97.4%<br>(96.0-97.9)	100.0%<br>(83.2-100)	96.0%<br>(79.6-100)	100.0%<br>(88.1-100)	100%<br>(83.2-100)	100.0%<br>(76.8-100)	99.1%<br>(94.9-100)
RsHr/Rs-TB	908/5395	68/256	176/832	171/1049	197/1008	206/1006	818/4151	7/88	16/288	31/371	24/266	12/231	90/1244
RsHr-TB%<br>(95%CI)	16.8%<br>(15.8-17.9)	26.6%<br>(21.3-32.4)	21.2%<br>(18.4-24.1)	16.3%<br>(14.1-18.7)	19.5%<br>(17.1-22.1)	20.5%<br>(18.0-23.1)	19.7%<br>(18.5-20.9)	8.0%<br>(3.3-15.7)	5.6%<br>(3.2-15.7)	8.4%<br>(5.7-11.7)	9.0%<br>(5.9-13.1)	5.2%<br>(2.7-8.9)	7.2%<br>(5.9-8.8)
**Genotypic DST**													
All gDST	10212	302	1840	2159	2305	2324	8930	24	297	407	291	263	1282
Any Rr-TB%<br>(95%CI)	5146;50.4%<br>(49.4-51.4)	212;70.2%<br>(64.7-75.3)	1061;57.7%<br>(55.3-60.0)	1123;52.0%<br>(49.9-54.1)	1348;58.5%<br>(56.4-60.5)	1283;55.2%<br>(53.2-57.2)	5027;56.3%<br>(55.2-57.3)	3;12.5%<br>(2.7-32.4)	28;9.4%<br>(6.4-13.3)	46;11.3%<br>(8.4-14.8)	25;8.6%<br>(5.6-12.4)	17;6.5%<br>(3.8-10.1)	119;9.3%<br>(7.7-11.0)
Any Hr-TB%<br>(95%CI)	4557;44.6%<br>(43.7-45.6)	194;64.2%<br>(58.5-69.6)	974;52.9%<br>(50.6-55.2)	1016;47.1%<br>(44.9-49.2)	1140;49.5%<br>(47.4-51.5)	1069;46.0%<br>(44.0-48.1)	4393;49.2%<br>(48.2-50.2)	4;16.7%<br>(4.7-37.4)	34;11.4%<br>(8.1-15.6)	63;15.5%<br>(12.1-19.4)	39;13.4%<br>(9.7-17.9)	24;9.1%<br>(5.9-13.3)	164;12.8%<br>(11.0-14.79
MDR/Rr-TB	4112/5146	179/212	881/1061	923/1123	1054/1348	971/1283	4008/5027	3/3	24/28	40/46	22/25	15/17	104/119
MDR%<br>(95%CI)	79.9%<br>(78.8-81.0)	84.4%<br>(78.8-89.0)	83.0%<br>(80.6-85.2)	82.2%<br>(79.8-84.3)	78.2%<br>(75.9-80.4)	75.7%<br>(73.2-78.0)	79.7%<br>(78.6-80.8)	100.0%<br>(29.2-100)	85.7%<br>(67.3-96)	87.0%<br>(73.7-95.1)	88.0%<br>(68.8-97.5)	88.2%<br>(63.6-98.5)	87.4%<br>(80.0-92.8)
RsHr/Rs-TB	445/5066	15/90	93/779	93/1036	86/957	98/1041	385/3903	1/21	10/269	23/361	17/266	9/246	60/1193
RsHr-TB%<br>(95%CI)	8.8%<br>(8.0-9.6)	16.7%<br>(9.6-26.0)	11.9%<br>(9.7.14.4)	9.0%<br>(7.3-10.9)	9.0%<br>(7.3-11.0)	9.4%<br>(7.7-11.4)	9.9%<br>(8.9-10.8)	4.8%<br>(0.1-23.8)	3.7%<br>(0.1-23.8)	6.4%<br>(1.8-6.7)	6.4%<br>(4.0-9.4)	3.7%<br>(3.8-100.0)	5.2%<br>(1.7-6.8)
**Phenotypic +Genotypic DST**													
All p+gDST	8787	295	1707	1860	1923	1766	7551	24	293	394	282	243	1236
Rr-TB%<br>(95%CI)	4303;49.0%<br>(47.9-50.0)	211;71.5%<br>(60.0-76.6)	998;58.5%<br>(56.1-60.8)	946;50.9%<br>(48.6-53.3)	1098;56.8%<br>(54.5-59.0)	938;53.1%<br>(50.8-55.5)	4185;55.4%<br>(54.3-56.5)	3;12.5%<br>(2.7-32.4)	34;11.6%<br>(8.2-15.8)	41;10.4%<br>(7.6-13.9)	24;8.5%<br>(5.5-13.0)	16;6.6%<br>(3.8-10.5)	118;9.5%<br>(8.0-11.3)
Any Hr-TB%<br>(95%CI)	4542;51.7%<br>(50.6-52.7)	222;75.3%<br>(69.9-80.1)	1062;62.2%<br>(59.9-64.5)	997;53.6%<br>(51.3-55.9)	1115;58.0%<br>(55.7-60.20	964;54.6%<br>(52.2-56.9)	4360;57.7%<br>(56.6-58.9)	4;16.7%<br>(4.7-37.4)	45;15.4%<br>(11.4-20)	63;16.0%<br>(12.5-20.0)	45;16.0%<br>(11.9-20.8)	25;10.3%<br>(6.8-14.8)	182;14.7%<br>(12.8-16.8)
Rr-TB/MDR	4078/4303	205/211	966/998	900/946	1034/1092	866/938	3971/4185	3/3	29/34	37/41	23/24	15/16	107/118
MDR%<br>(95%CI)	94.8%<br>(94.1-95.4)	97.2%<br>(93.9-98.9)	96.8%<br>(95.5-98.0)	95.1%<br>(93.6-96.4)	94.7%<br>(93.2-95.9)	92.3%<br>(90.4-93.9)	94.9%<br>(94.2-95.5)	100.0%<br>(29.2-100)	85.3%<br>(68.9-95)	90.2%<br>(76.9-97.3)	95.8%<br>(78.9-100)	93.8%<br>(69.8-99.8)	90.7%<br>(83.9-95.2)
RsHr/Rs-TB	464/4484	17/84	96/709	97/914	81/831	98/828	389/3366	1/21	16/259	26/353	22/258	10/227	75/118
RsHr-TB%<br>(95%CI)	10.3%<br>(9.5-11.3)	20.2%<br>(12.2-30.4)	13.5%<br>(11.1-16.2)	10.6%<br>(8.6-12.8)	9.7%<br>(7.8-12.0)	11.8%<br>(9.7-14.2)	11.6%<br>(10.5-12.7)	4.8%<br>(0.1-23.8)	6.2%<br>(3.6-9.8)	7.4%<br>(4.9-10.6)	8.5%<br>(5.4-12.6)	4.4%<br>(2.1-8.0)	6.7%<br>(5.4-7.2)

R-Rifampicin, H-Isoniazid, r-Resistant, s-susceptible, DST-Drug susceptibility testing, p-Phenotypic; g-Genotypic
