# Supplementary material for: Isoniazid resistance profile and associated levofloxacin and pyrazinamide resistance in rifampicin resistant and sensitive isolates/from pulmonary and extrapulmonary tuberculosis patients in Pakistan: A laboratory based surveillance study 2015-19
Source: PLoS One. 2020 Sep 23;15(9):e0239328. doi: 10.1371/journal.pone.0239328 (PMC7511002; doi:10.1371/journal.pone.0239328)
Supplement: S4 Table — RMP-rifampicin, INH-isoniazid, R-resistant, S-sensitive, pDST-phenotypic, drug susceptibility testing; gDST-Genotypic drug susceptibility testing. (PDF) [file pone.0239328.s004.pdf]

S4-Table: Rifampicin and isoniazid resistance trend and correlation between results of phenotypic and genotypic drug susceptibility testing, National TB reference Laboratory, Pakistan 2015-2019.

	Annual trend					Total	Previous TB Treatment		Disease site	
	2015	2016	2017	2018	2019	2016-19	New	PT	PTB	EPTB
**All Isolates**	319	2000	2254	2205	2009	8787	4713	4074	7551	1236
Concordant Result										
RMP & INH	247<br>77.4%	1599<br>80.0%	1879<br>83.4%	1791<br>81.2%	1601<br>79.7%	7117<br>81.0%	4044<br>85.8%	3073<br>75.4%	5994<br>79.4%	1123<br>90.9%
RMP	293<br>91.8%	1859<br>93.0%	2102<br>93.3%	2025<br>91.8%	1830<br>91.1%	8109<br>92.3%	4486<br>95.2%	3623<br>88.9%	6909<br>91.5%	1200<br>97.1%
INH	284<br>89.0%	1790<br>89.5%	2098<br>93.1%	2041<br>92.6%	1858<br>92.5%	8071<br>91.9%	4463<br>94.7%	3608<br>88.6%	6888<br>91.2%	1201<br>97.2%
**RMP-Resistant**										
All by pDST and/or gDST	214	1032	987	1116	954	4303	1166	3137	4185	118
Concordant results	188<br>88%	891<br>86%	835<br>85%	936<br>84%	775<br>81%	3625<br>84.2%	939<br>80.5%	2686<br>85.6%	3543<br>84.7%	82<br>69.5%
RMP-Sensitive-pDST	22<br>10.3%	101<br>9.8%	127<br>12.9%	161<br>14.4%	159<br>16.7%	570<br>13.2%	187<br>16.0%	383<br>12.2%	543<br>13.0%	27<br>22.9%
RMP-Sensitive-gDST	4<br>1.9%	40<br>3.9%	25<br>2.5%	19<br>1.7%	20<br>2.1%	108<br>2.5%	40<br>3.4%	68<br>2.2%	99<br>2.4%	9<br>7.6%
**INH-Resistant**										
All pDST and/or gDST	226	1107	1060	1160	989	4542	1399	3143	4360	182
Concordant results	191<br>84.5%	897<br>81.0%	904<br>85.3%	996<br>85.9%	838<br>84.7%	3826<br>84.2%	1149<br>82.1%	2677<br>85.2%	3679<br>84.4%	147<br>80.8%
INH-Sensitive-pDST	3<br>1.3%	22<br>2.0%	18<br>1.7%	8<br>0.7%	5<br>0.5%	56<br>1.2%	35<br>2.5%	21<br>0.7%	47<br>1.1%	9<br>4.9%
INH-sensitive-gDST	32<br>14.2%	188<br>17.0%	138<br>13.0%	156<br>13.4%	146<br>14.8%	660<br>14.5%	215<br>15.4%	445<br>14.2%	634<br>14.5%	26<br>14.3%
**Multi-drug Resistance**										
All by pDST and/or gDST	208	995	937	1057	881	4078	1082	2996	3971	107
Concordant results<br>MDR-pDST and gDST	160<br>76.9%	743<br>74.7%	735<br>78.4%	805<br>76.2%	654<br>74.2%	3097<br>75.9%	814<br>75.2%	2283<br>76.2%	3017<br>76.0%	80<br>74.8%
**pDST (RMP&INH)**										
RMP-R/INH-R	187<br>89.9%	898<br>90.3%	843<br>90.0%	933<br>88.3%	771<br>87.5%	3632<br>89.1%	953<br>88.1%	2679<br>89.4%	3542<br>89.2%	90<br>84.1%
RMP-S/INH-R	19<br>9.1%	80<br>8.0%	85<br>9.1%	122<br>11.5%	105<br>11.9%	411<br>10.1%	113<br>10.4%	298<br>9.9%	400<br>10.1%	11<br>10.3%
RMP-R/INH-S	1<br>0%	5<br>1%	1<br>0%	2<br>0%	0<br>0%	9<br>0%	1<br>0%	8<br>0%	9<br>0%	0<br>0%
RMP-S/INH-S	1<br>0.5%	12<br>1.2%	8<br>0.9%	0<br>0.0%	5<br>0.6%	26<br>0.6%	15<br>1.4%	11<br>0.4%	20<br>0.5%	6<br>5.6%
**gDST(RMP &INH)**										
RMP-R/INH-R	178<br>85.6%	827<br>83.1%	819<br>87.4%	909<br>86.0%	751<br>85.2%	3484<br>85.4%	928<br>85.8%	2556<br>85.3%	3388<br>85.3%	96<br>89.7%
RMP-S/INH-R	3<br>1.4%	19<br>1.9%	13<br>1.4%	14<br>1.3%	17<br>1.9%	66<br>1.6%	20<br>1.8%	46<br>1.5%	64<br>1.6%	2<br>1.9%
RMP-R/INH-S	26<br>12.5%	132<br>13.3%	93<br>9.9%	129<br>12.2%	110<br>12.5%	490<br>12.0%	115<br>10.6%	375<br>12.5%	487<br>12.3%	3<br>2.8%
RMP-S/INH-S	1<br>0.5%	17<br>1.7%	12<br>1.3%	5<br>0.5%	3<br>0.3%	38<br>0.9%	19<br>1.8%	19<br>0.6%	32<br>0.8%	6<br>5.6%

RMP-Rifampicin, INH-Isoniazid, R-Resistant, S-Sensitive, pDST-Phenotypic Drug susceptibility testing; gDST-Genotypic Drug susceptibility testing
