# Supplementary material for: Isoniazid resistance profile and associated levofloxacin and pyrazinamide resistance in rifampicin resistant and sensitive isolates/from pulmonary and extrapulmonary tuberculosis patients in Pakistan: A laboratory based surveillance study 2015-19
Source: PLoS One. 2020 Sep 23;15(9):e0239328. doi: 10.1371/journal.pone.0239328 (PMC7511002; doi:10.1371/journal.pone.0239328)
Supplement: S5 Table — INH-isoniazid. (PDF) [file pone.0239328.s005.pdf]

S5-Table: Molecular characterization of Isoniazid resistance in Mtb isolates, from pulmonary and extrapulmonary TB patients stratified by rifampicin resistance and history of TB treatment, National TB reference laboratory, Pakistan, 2015-2019

	Pulmonary Tuberculosis							Extrapulmonary Tuberculosis						
**INH genetic profile**	New			Previously Treated			p-value	New			Previously treated			p-value
	n	%	(95% CI)	n	%	(95% CI)		n	%	(95% CI)	n	%	(95% CI)	
**Rifampicin Resistant TB**														
Total tested (n)	**1086**			**3099**				**80**			**38**			
INH-Sensitive (gWTpWT)	74	6.8%	(5.4 -8.5)	140	4.5%	(3.8-5.3)	0.003	10	12.5%	(6.2-22.8)	1	2.6%	(0.07-13.8)	0.084
INH-Resistant (All)	1012	93.2%	(91.5-94.6)	2959	95.5%	(94.7-96.2)	0.003	70	87.5%	(78.2-93.8)	37	97.4%	(86.2-99.9)	0.084
• gWTpNWT	127	12.5%	(10.6-14.7)	392	13.2%	(12.0-14.5)	0.555	7	10.0%	(4.1-19.5)	2	5.4%	(0.7-18.2)	0.403
• *inh*A mutation	80	7.9%	(6.3-9.7)	220	7.4%	(6.5-8.4)	0.591	5	7.1%	(2.4-15.9)	6	16.2%	(6.2-32.0)	0.124
• *kat*G mutation	779	77.0%	(74.3-79.5)	2248	76.0%	(74.4-77.5)	0.505	56	80.0%	(68.7-88.6)	29	78.4%	(61.8-90.2)	0.084
• Double Mutation	26	2.6%	(1.7-3.7)	99	3.3%	(2.8-4.1)	0.253	2	2.9%	(0.3-9.9)	0	0.0%	(0.0-0.0)	0.289
**Rifampicin Susceptible TB**														
Total tested	**2489**			**877**				**1058**			**60**			
INH-Sensitive (gWTpWT)	2244	90.2%	(88.9-91.2)	733	83.6%	(81.0-86.0)	**<0.01**	986	93.2%	(91.5-94.6)	57	95.0%	(86.1-99.0)	0.588
INH-Resistant (All)	245	9.8%	(8.7-11.1)	144	16.4%	(14.0-19.0)	**<0.01**	72	6.8%	(5.4-8.5)	3	5.0%	(1.0-13.9)	0.588
• gWTpNWT	64	26.1%	(20.7-32.1)	51	35.4%	(27.6-43.8)	**<0.01**	17	23.6%	(14.4-35.1)	0	0.0%	(0.0-0.0)	<0.01
• *inh*A mutation	76	31.0%	(25.3-37.2)	39	27.1%	(20.0-35.1)	0.03	24	33.3%	(22.7-45.4)	1	33.3%	(0.8-90.6)	1.00
• *kat*G mutation	105	42.9%	(36.6-49.3)	53	36.8%	(28.9-45.2)	0.002	31	43.1%	(31.4-55.3)	2	66.7%	(9.4-99.2)	<0.01
• Double Mutation	0	0.0%	(0.0-0.0)	1	0.7%	(0.0-3.8)	<0.01	0	0.0%	(0.0-0.0)	0	0.0%	(0.0-0.0)	NA
