# Supplementary material for: Isoniazid resistance profile and associated levofloxacin and pyrazinamide resistance in rifampicin resistant and sensitive isolates/from pulmonary and extrapulmonary tuberculosis patients in Pakistan: A laboratory based surveillance study 2015-19
Source: PLoS One. 2020 Sep 23;15(9):e0239328. doi: 10.1371/journal.pone.0239328 (PMC7511002; doi:10.1371/journal.pone.0239328)
Supplement: S6 Table — RMP-rifampicin, INH-isoniazid, PT-Previously treated. (PDF) [file pone.0239328.s006.pdf]

S6-Table: Annual trend of genotypic profile of isoniazid resistance associated with rifampicin resistant and sensitive Mtb isolates from new and previously treated TB patients, National TB Reference laboratory, Pakistan 2015-19

	2015			2016			2017			2018			2019		
**Rifampicin resistant**	**n**	**%**	**95%CI**	**n**	**%**	**95%CI**	**n**	**%**	**95%CI**	**n**	**%**	**95%CI**	**n**	**%**	**95%CI**
**All-RMP-resistant isolates tested**	**214**			**1032**			**987**			**1116**			**954**		
INH-resistant	208	97.2%	94.0-99.0	995	96.4%	95.1-97.5	937	94.9%	93.4-96.2	1057	94.7%	93.2-96.0	881	92.3%	90.5-94.0
gWTpNWT	27	13.0%	8.7-18.3	149	15.0%	12.8-17.3	105	11.2%	9.3-13.4	134	12.7%	10.7-14.8	113	12.8%	10.7-15.2
katG mutation	153	73.6%	67.0-79.4	745	74.9%	72.1-77.5	746	79.6%	76.9-82.2	797	75.4%	72.7-78.0	671	76.2%	73.2-78.9
inhA mutation	21	10.1%	6.4-15.0	67	6.7%	5.3-08.5	63	6.7%	5.2-8.5	94	8.9%	7.2-10.8	66	7.5%	5.8-9.4
Double Mutation	7	3.4%	1.4-6.8	34	3.4%	2.4-4.7	23	2.5%	1.6-3.7	32	3.0%	2.1-4.2	31	3.5%	2.4-5.0
**New-RMP-resistant isolates tested**	**22**			**167**			**298**			**338**			**341**		
INH-resistant	21	95.5%	77.2-99.9	155	92.8%	87.8-96.2	278	93.3%	89.8-95.9	317	93.8%	90.7-96.1	311	91.2%	87.7-94.0
gWTpNWT	3	14.3%	3.0-36.3	17	11.0%	6.5-17.0	37	13.3%	9.5-17.9	36	11.4%	8.1-15.4	41	13.2%	9.6-17.5
katG mutation	17	81.0%	58.1-94.6	123	79.4%	72.1-85.4	210	75.5%	70.0-80.5	242	76.3%	71.3-80.9	243	78.1%	73.1-82.6
inhA mutation	1	4.8%	00.1-23.8	10	6.5%	3.1-11.5	23	8.3%	5.3-12.2	30	9.5%	6.5-13.2	21	6.8%	4.2-10.1
Double Mutation	0	0.0%	0	5	3.2%	1.1-7.4	8	2.9%	1.3-5.6	9	2.8%	1.3-5.3	6	1.9%	0.7-4.2
**PT- RMP-resistant-isolates tested**	**192**			**865**			**689**			**778**			**613**		
INH-resistant	187	97.4%	94.0-99.1	840	97.1%	95.8-98.1	659	95.6%	93.8-97.0	740	95.1%	93.4-96.5	570	93.0%	90.7-94.9
gWTpNWT	24	12.8%	8.4-18.5	132	15.7%	13.3-18.9	68	10.3%	8.1-12.9	98	13.2%	10.9-15.9	72	12.6%	10.0-15.6
katG mutation	136	72.7%	65.7-79.0	622	74.0%	70.9-77.0	536	81.3%	78.1-84.2	555	75.0%	71.7-78.1	428	75.1%	71.3-78.6
inhA mutation	20	10.7%	6.7-16.0	57	6.8%	5.2-8.7	40	6.1%	4.4-8.2	64	8.6%	6.7-10.9	45	7.9%	5.8-10.4
Double Mutation	7	3.7%	1.5-7.6	29	3.5%	2.3-4.9	15	2.3%	1.3-3.7	23	3.1%	2.0-4.6	25	4.4%	2.9-6.4
**Rifampicin sensitive**															
**All RMP-sensitive isolates tested**	**105**			**968**			**1267**			**1089**			**1055**		
INH-resistant	18	17.1%	10.5-.25.7	112	11.6%	9.6-13.8	123	9.7%	8.1-11.5	103	9.5%	7.8-11.4	108	10.2%	8.5-12.2
gWTpNWT	5	27.8%	9.7-53.5	39	34.8%	26.1-44.4	33	26.8%	19.2-35.6	22	21.4%	14.0-30.5	33	30.6%	22.1-40.2
katG mutation	7	38.9%	17.3-64.3	48	42.9%	33.5-52.6	52	42.3%	33.4-51.5	43	41.7%	32.1-51.9	41	38.0%	28.8-47.8
inhA mutation	5	27.8%	9.7-53.5	25	22.3%	15.0-31.2	38	30.9%	22.9-39.9	38	36.9%	27.6-47.0	34	31.5%	22.9-41.1
Double Mutation	1	5.6%	0.1-27.3	0	0%	0.0-0.0	0	0%	0.0-0.0	0	0.0%	0.0-0.0	0	0.0%	0.0-0.0
**All RMP-sensitive-isolates tested**	**83**			**723**			**1075**			**884**			**782**		
INH-resistant	11	13.3%	6.8-22.5	62	8.6%		96	8.9%	6.6-10.9	79	8.9%	7.1-11.0	69	8.8%	6.9-11.0
gWTpNWT	3	27.3%	6.0-61.0	14	22.6%	12.9-35.0	22	22.9%	15.6-34.0	19	24.1%	15.1-35.0	23	33.3%	22.4-45.7
katG mutation	6	54.5%	23.4-83.3	29	46.8%	34.0-59.9	43	44.8%	36.3-57.4	31	39.2%	28.4-50.9	27	39.1%	27.6-51.6
inhA mutation	2	18.2%	2.3-51.8	19	30.6%	19.6-43.7	31	32.3%	24.2-44.3	29	36.7%	26.1-48.3	19	27.5%	17.4-39.6
Double Mutation	0	0.0%	0.0-	0	0.0%	0.0-5.8	0	0.0%	0.0-3.9	0	0.0%	0.0-4.6	0	0.0%	0.0-5.2
**PT-RMP-sensitive-Isolates tested**	**22**			**245**			**192**			**205**			**273**		
INH-resistant	7	31.8%	13.9-54.9	50	20.4%	15.5-26.0	27	14.1%	9.5-19.8	24	11.7%	9.5-19.8	39	14.3%	10.4-19.0
gWTpNWT	2	28.6%	3.7-71.0	25	50.0%	35.5-64.5	11	40.7%	22.4-61.2	3	12.5%	2.7-32.4	10	25.6%	13.0-42.1
katG mutation	1	14.3%	0.4-57.9	19	38.0%	24.7-52.8	9	33.3%	16.5-54.0	12	50.0%	29.1-70.9	14	35.9%	21.2-52.8
inhA mutation	3	42.9%	1.0-81.6	6	12.0%	4.5-24.3	7	25.9%	11.1-46.3	9	37.5%	18.8-59.4	15	38.5%	23.4-55.4
