# Supplementary material for: Isoniazid resistance profile and associated levofloxacin and pyrazinamide resistance in rifampicin resistant and sensitive isolates/from pulmonary and extrapulmonary tuberculosis patients in Pakistan: A laboratory based surveillance study 2015-19
Source: PLoS One. 2020 Sep 23;15(9):e0239328. doi: 10.1371/journal.pone.0239328 (PMC7511002; doi:10.1371/journal.pone.0239328)
Supplement: S7 Table — n-Number of isolates resistant to Levofloxacin and/or Pyrazinamide, N- Number of isolates tested INH-isoniazid. (PDF) [file pone.0239328.s007.pdf]

S7-Table; Levofloxacin and Pyrazinamide resistance associated with isoniazid resistance in rifampicin resistant and sensitive Mtb isolates from pulmonary and extrapulmonary TB patients stratified by history of TB treatment, National TB Reference laboratory, Pakistan, 2015-19

	**Rifampicin Resistant**								**Rifampicin Sensitive**							
	Pulmonary Tuberculosis				Extrapulmonary Tuberculosis				Pulmonary Tuberculosis				Extrapulmonary Tuberculosis			
	New		Previously treated		New		Previously treated		New		Previously treated		New		Previously treated	
**INH genetic profile**	n<br>N	%R<br>(95% CI)	n<br>N	%R<br>(95% CI)	n<br>N	%R<br>(95% CI)	n<br>N	%R<br>(95% CI)	n<br>N	%R<br>(95% CI)	n<br>N	%R<br>(95% CI)	n<br>N	%R<br>(95% CI)	n<br>N	%R<br>(95% CI)
**LEVOFLOXACIN**																
All Isolates	513	47.3%	1484	47.9%	39	48.8%	14	36.8%	330	13.3%	156	17.8%	79	7.5%	1	1.7%
	1085	(44.3-50.3)	3098	(46.5-1-50.0)	80	(34.4-60.1)	38	(21.8-54.0)	2488	(12.0-14.7)	877	15.3-20.5)	1057	(6.0-9.2)	60	(0.04-8.9)
INH-Sensitive (gWTpWT)	18	24.3%	33	23.6%	1	10.0%	0	0.0%	283	12.6%	105	14.3%	74	7.5%	1	1.8%
	74	(15.1-35.7)	140	(16.8-31.5)	10	(0.3-44.5)	1	(0.0)	2243	(11.3-14.1)	733	(11.9-17.1)	985	(5.9-9.3)	57	(0.04-9.4)
INH-Resistant (ALL)	495	49.0%	1451	49.1%	38	54.3%	14	37.8%	47	19.2%	51	35.4%	5	6.9%	0	0.00%
	1011	(45.8-51.1)	2958	(47.2-50.9)	70	(41.9-66.3)	37	(22.5-55.2)	245	(14.4-24.7)	144	(27.6-43.8)	72	(2.3-15.5)	3	0
• gWTpNWT	49	38.6%	164	41.9%	3	42.9%	2	100.0%	19	29.7%	19	37.3%	1	5.9%	0/0	0.0%
	127	(30.1-47.6)	391	(37.0-47.0)	7	(10.0-81.6)	2	(15.8-100.0)	64	(18.9-42.4)	51	(24.1-51.9)	17	(0.1-28.7)	0/0	(0.0)
• *inhA* mutation	33	41.3%	105	47.7%	2	40.0%	0	0.0%	12	15.8%	12	30.8%	1	4.2%	0	0.0%
	80	(30.4-52.8)	220	(41.0-54.5)	5	(5.3-85.3)	6	(0.0)	76	(8.4-26.0)	39	(17.0-47.6)	24	(0.1-21.1)	1	(0.0)
• *KatG* mutation	397	51.0%	1107	49.2%	32	57.1%	12	41.4%	16	15.2%	20	37.7%	3	9.7%	0	0.0%
	778	(47.5-42.6)	2248	(47.2-51.3)	56	(43.2-70.3)	29	(23.5-61.1)	105	(9.0-23.7)	53	(24.8-52.1)	31	(2.0-25.8)	2	(0.0)
• Double Mutation	16	61.5%	75	75.8%	1	50.0%	0	0.0%	0	0.0%	0	0.0%	0	0.0%	0	0.0%
	26	(40.6-79.8)	99	(61.1-83.8)	2	(1.3-98.7)	0	(0.0)	0	(0.0)	1	(0.0)	0	(0.0)	0	(0.0)
**PYRAZINAMIDE**																
All Isolates	427	43.5%	1228	45.2%	35	50.0%	23	67.6%	74	3.3%	40	5.1%	29	3.0%	0	0.0%
	981	(40.4-46.7)	2715	(43.3-47.1)	70	(37.8-62.2)	34	(49.5-82.6)	2266	(26.0-4.1)	782	(3.7-6.9)	971	(2.0-4.3)	48	(0.0)
INH-Sensitive (gWTpWT)	3	4.3%	10	7.6%	0	0.0%	0	0.0%	50	2.4%	22	3.3%	23	2.5%	0	0.0%
	70	(0.9-12.0)	132	(3.7-13.5)	9	(0.0)	1	(0.0)	2046	(1.8-3.2)	658	(2.1-5.0)	906	(1.6-3.8)	45	(0.0)
INH-Resistant (ALL)	424	46.5%	1218	47.2%	35	57.4%	23	69.7%	24	10.9%	18	14.5%	6	9.2%	0	0.00%
	911	(43.3-49.8)	2583	(45.2-49.1)	61	(44.1-70.0)	33	(51.3-84.4)	220	(7.1-15.8)	24	(8.8-22.0)	65	(3.5-9.0)	3	0
• gWTpNWT	31	28.2%	102	30.4%	2	40.0%	0	0.0%	9	15.0%	7	17.9%	1	6.7%	0	0.0%
	110	(20.0-37.6)	335	(25.6-35.7)	5	(5.3-85.3)	1	(0.0)	60	(71.0-26.6)	39	(7.5-33.5)	15	(0.2-31.9)	0	(0.0)
• *inhA* mutation	21	28.4%	63	32.8%	0	0.0%	4	66.7%	3	4.4%	2	5.4%	5	21.7%	0	0.0%
	74	(18.5-40.0)	192	(26.2-39.9)	4	(0.0)	6	(22.2-95.7)	68	(0.9-12.4)	37	(6.6-18.2)	23	(7.5-43.7)	1	(0.0)
• *KatG* mutation	360	51.0%	994	50.5%	32	64.0%	19	73.1%	12	13.0%	9	19.1%	0	0.0%	0	0.0%
	706	(47.2-54.7)	1970	(48.2-52.7)	50	(49.2-77.1)	26	(52.2-88.4)	92	(6.9-21.7)	47	(9.1-33.3)	27	(0.0)	2	(0.0)
• Double Mutation	12	57.1%	59	68.6%	1	50.0%	0	0.0%	0	0.0% (0.0)	0	0.0%	0	0.0%	0	0.0%
	21	(34.0-78.2)	86	(57.7-78.2)	2	(12.6-98.7)	0	(0.0)	0	0.0% (0.0)	1	(0.0)	0	(0.0)	0	(0.0)
**COMBINED LEVOFLOXACIN AND PYRAZINAMIDE**																
All Isolates	256	26.1%	725	26.7%	22	31.4%	9	26.5%	10	0.4%	21	2.7%	2	0.2%	0	0.0%
	981	(8.8-22.0)	2715	(25.0-28.1)	70	(20.9-43.6)	34	(12.9-44.4)	2265	(0.2-0.8)	782	(1.7-40.1)	970	(0.02-0.7)	48	(0.0)
INH-Sensitive (gWTpWT)	2	2.9%	4	3.0%	0	0.0%	0	0.0%	3	0.1%	6	0.9%	2	0.2%	0	0.0%
	70	(0.3-9.9)	132	(0.8-75.8)	9	(0.0)	1	(0.0)	2045	(0.03-0.4)	658	(0.3-2.0)	905	0(0.02-0.8)	45	(0.0)
INH-Resistant(ALL)	254	27.9%	721	27.9%	22	36.1%	9	27.3%	7	3.2%	15	12.1%	0	0.0%	0	0.00%
	911	(25.0-30.9)	2583	(26.2-29.7)	61	(24.1-49.4)	33	(13.3-45.5)	220	(1.3-13.9)	124	(6.9-19.2)	65	0	3	0
• gWTpNWT	11	10.0%	45	13.4%	1	20.0%	0	0.0%	3	5.0%	7	17.9%	0	0.0%	0	0.0%
	110	(50.1-17.2)	335	(10.0-17.6)	5	(0.5-71.6)	1	(0.0)	60	(1.0-13.9)	39	(7.5-33.5)	15	(0.0)	0	(0.0)
• *inhA* mutation	12	16.2%	43	22.4%	0	0.0%	0	0.0%	0	0.0%	1	2.7%	0	0.0%	0	0.0%
	74	(8.7-26.6)	192	(16.7-29.0)	4	(0.0)	6	(0.0)	68	(0.0)	37	(0.07-14.2)	23	(0.0)	1	(0.0)
• *KatG* mutation	221	31.3%	584	29.6%	20	40.0%	9	34.6%	4	4.3%	7	14.9%	0	0.0%	0	0.0%
	706	(27.9-34.9)	1970	(27.6-31.7)	50	(26.4-54.8)	26	(17.2-55.7)	92	(1.1-10.8)	47	(6.2-28.3)	27	(0.0)	2	(0.0)
• Double Mutation	10	47.6%	49	57.0%	1	50.0%	0	0.0%	0	0.0%	0	0.0%	0	0.0%	0	0.0%
	21	(25.7-70.2)	86	(45.8-67.8)	2	(1.3-98.7)	0	(0.0)	0	(0.0)	1	(0.0)	0	(0.0)	0	(0.0)

n=Number of isolates resistant to Levofloxacin and/or Pyrazinamide, N = Number of isolates tested
