# Supplementary material for: Isoniazid resistance profile and associated levofloxacin and pyrazinamide resistance in rifampicin resistant and sensitive isolates/from pulmonary and extrapulmonary tuberculosis patients in Pakistan: A laboratory based surveillance study 2015-19
Source: PLoS One. 2020 Sep 23;15(9):e0239328. doi: 10.1371/journal.pone.0239328 (PMC7511002; doi:10.1371/journal.pone.0239328)
Supplement: S8 Table — LFX-Levofloxacin, PZA-Pyrazinamide, n = Number of isolates resistant, N = Number of isolates tested. (PDF) [file pone.0239328.s008.pdf]

S8-Table; Annual trend of levofloxacin and pyrazinamide resistance associated with isoniazid resistance in rifampicin resistant and susceptible Mtb isolates from pulmonary and extrapulmonary TB patients, National TB Reference laboratory, Pakistan, 2015-19

	Rifampicin-resistant isoniazid- Resistant (RrHr-TB)						Rifampicin sensitive isoniazid resistant(RsHr-TB)					
	2015	2016	2017	2018	2019	Total	2015	2016	2017	2018	2019	Total
**Pulmonary TB**												
**Levofloxacin Resistance**												
Isolates tested (N)	205	964	900	1034	866	3969	17	96	97	81	98	389
LFX-Resistant (n)	109	498	431	498	410	1946	4	27	19	19	29	98
LFX-Resistant (%)	53.2%	51.7%	47.9%	48.2%	47.3%	49.0%	23.5%	28.1%	19.6%	23.5%	29.6%	25.2%
95% CI	46.1-60.2	48.5-54.9	44.6-51.2	45.1-51.3	44.0-50.7	47.5-50.6	6.8-49.9	19.4-38.2	12.2-28.9	14.8-34.2	20.8-39.7	21.0-29.8
**Pyrazinamide Resistance**												
Isolates tested (N)	194	692	834	961	813	3494	15	66	93	79	91	344
PZA-Resistant (n)	114	350	401	447	330	1642	1	15	11	6	9	42
PZA Resistant (%)	58.8%	50.6%	48.1%	46.5%	40.6%	47.0%	6.7%	22.7%	11.8%	7.6%	9.9%	12.2%
95% CI	51.5.65.8	46.8-54.4	44.6-51.5	43.3-49.7	37.2-44.1	45.3-48.7	0.2-31.9	13.3-34.7	6.1-20.2	2.8-15.8	4.6-17.9	8.9-16.1
**Extrapulmonary TB**												
**Levofloxacin Resistance**												
Isolates tested (N)	3	29	37	23	15	107	1	16	26	22	10	75
LFX-Resistant (n)	2	10	17	9	10	48	1	1	0	1	2	5
LFX-Resistance (%)	66.7%	34.5%	45.9%	39.1%	66.7%	44.9%	100.0%	6.3%	0.0%	4.5%	20.0%	6.7%
95% CI	9.4-99.2	17.9-54.3	29.5-63.1	19.7-61.5	38.4-88.2	35.2-54.8	2.5-100.0	0.2-30.2	0	0.1-22.8	2.5-55.6	2.2-14.9
**Pyrazinamide Resistance**												
Isolates tested (N)	3	21	34	21	15	94	1	14	25	18	10	68
PZA-Resistant (n)	2	11	20	13	12	58	0	2	3	0	1	6
PZA-Resistant (%)	66.7%	52.4%	58.8%	61.9%	80.0%	61.7%	0.0%	14.3%	12.0%	0.0%	10.0%	8.8%
95% CI	9.4-99.2	29.8-74.3	40.7-75.4	38.4-81.9	51.9-95.7	51.1-71.5	0	1.8-42.8	2.5-31.2	0	0.3-44.5	3.3-18.2
