# Supplementary figures and images for: Isoniazid resistance profile and associated levofloxacin and pyrazinamide resistance in rifampicin resistant and sensitive isolates/from pulmonary and extrapulmonary tuberculosis patients in Pakistan: A laboratory based surveillance study 2015-19
Source: PLoS One. 2020 Sep 23;15(9):e0239328. doi: 10.1371/journal.pone.0239328 (PMC7511002; doi:10.1371/journal.pone.0239328)

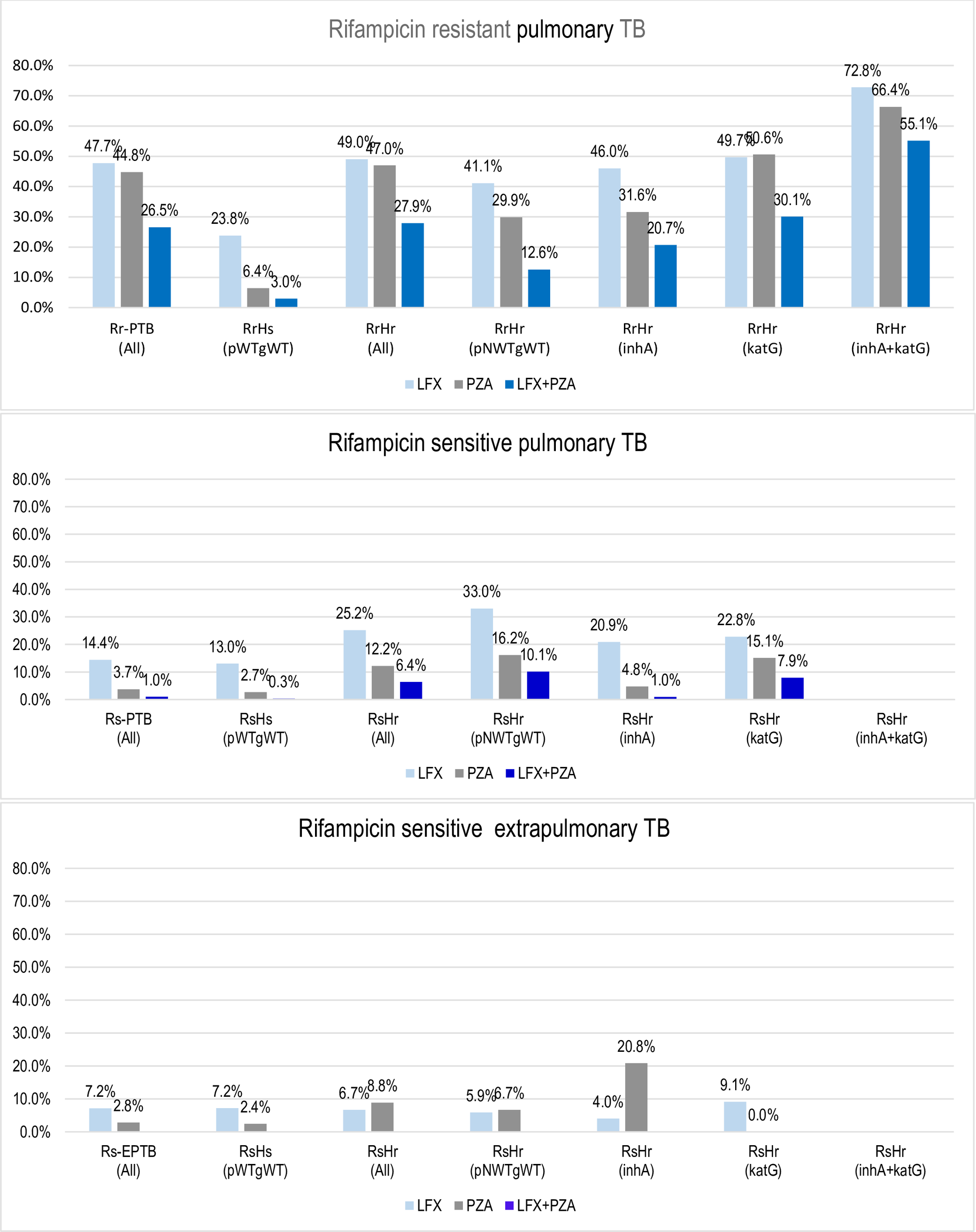

Supplement: S1 Fig — National TB reference Laboratory, Pakistan 2015–2019. Rr-Rifampicin resistant, Rs-Rifampicin sensitive, Hr-Isoniazid resistance, Hs-Isoniazid sensitive, isoniazid sensitive. (TIF) [file pone.0239328.s009.tif]
